# The Relationship Between Spin Crossover (SCO) Behaviors, Cation and Ligand Motions, and Intermolecular Interactions in a Series of Anionic SCO Fe(III) Complexes with Halogen-Substituted Azobisphenolate Ligands

**DOI:** 10.3390/molecules29225473

**Published:** 2024-11-20

**Authors:** Mai Hirota, Suguru Murata, Takahiro Sakurai, Hitoshi Ohta, Kazuyuki Takahashi

**Affiliations:** 1 Department of Chemistry, Graduate School of Science, Kobe University, 1-1, Rokkodai-cho, Nada-ku, Kobe 657-8501, Hyogo, Japan; 238s219s@stu.kobe-u.ac.jp (M.H.); murata.sgr@gmail.com (S.M.); 2 Research Facility Center for Science and Technology, Kobe University, 1-1, Rokkodai-cho, Nada-ku, Kobe 657-8501, Hyogo, Japan; tsakurai@kobe-u.ac.jp; 3 Molecular Photoscience Research Center, Kobe University, 1-1, Rokkodai-cho, Nada-ku, Kobe 657-8501, Hyogo, Japan; hohta@kobe-u.ac.jp

**Keywords:** spin crossover, Fe(III) complex, anionic complex, 2,2′-azobisphenolate ligand, halogen substituent effect, cooperativity, rotational motion, noncovalent interaction, QTAIM analysis

## Abstract

To investigate the halogen substitution effect on the anionic spin crossover (SCO) complexes, azobisphenolate ligands with 5,5′-dihalogen substituents from fluorine to iodine were synthesized, and their anionic Fe^III^ complexes **1F**, **1Cl**, **1Br**, and **1I** were isolated. The temperature dependence of magnetic susceptibility and crystal structure revealed that **1F**, **1Cl**, and **1Br** are all isostructural and exhibit SCO with the rotational motion of the cation and ligands, whereas **1I** shows incomplete SCO. Note that **1Cl** and **1Br** showed irreversible and reversible cooperative SCO transitions, respectively. Short intermolecular contacts between the Fe^III^ complex anions were found despite Coulomb repulsions for all the complexes. The topological analysis of the electron density distributions revealed the existence of X···X halogen bonds, C–H···X, C–H···N, and C–H···O hydrogen bonds, and C–H···π interactions are evident. The dimensionality of intermolecular interactions is suggested to be responsible for the cooperative SCO transitions in **1Cl** and **1Br**, whereas the disorder due to the freezing of ligand rotations in **1Cl** is revealed to inhibit the SCO cooperativity.

## 1. Introduction

Spin crossover (SCO) is the phenomenon in which the spin state of a central metal ion with a d^4^–d^7^ configuration in an octahedral transition metal complex changes between the low-spin (LS) and high-spin (HS) states in response to external stimuli such as temperature, pressure, light, and magnetic fields. SCO has attracted much attention as an externally responsive molecular switch because it changes not only the magnetism associated with the spin state change but also the color and coordination structure [1,2,3,4,5,6]. Taking advantage of these features, their applications have been devoted to displays [7], sensors [8,9], actuators [10,11,12], and patterned thin films [13], as well as for switching various physical properties such as conducting [14,15,16,17], magnetic [18,19,20,21], dielectric [22], and optical properties [23,24,25].

A SCO transition behavior in a solid is affected by various factors such as counterions, solvated molecules [26], substituents of ligands [27,28,29,30,31,32,33], and crystal polymorphs [34]. The SCO transition enthalpy in a solid is known to be divided into the SCO transition enthalpy of molecules and the lattice enthalpy difference on the SCO transition [26,35]. Since the steric and electronic effects of ligand substituents contribute to both enthalpy differences, it is very difficult to determine either the steric or electronic effect of ligand substitution quantitatively in the solid state. To clarify the electronic effect on ligand substitution, Halcrow et al. investigated the SCO behavior in the solution state and revealed the linear relationship between the Hammett constants of substituents and the transition temperatures [36]. On the other hand, although it is very difficult to determine the electronic effect in the crystalline state, Park et al. successfully demonstrated that the electron-withdrawing effect of halogen substituents is responsible for the decrease in SCO transition enthalpy and the increase in SCO transition temperature [29]. Probably this is because all the complexes with halogen substituents are isostructural due to small differences in molecular and crystal structures. Although there have been a large number of reports about the substituent effects of SCO complexes, most of them have been studied for neutral or cationic SCO complexes. Since the number of anionic SCO complexes is very limited, to the best of our knowledge, there is no systematic investigation of substituent effects of the ligand for anionic SCO complexes.

**Figure 1:**
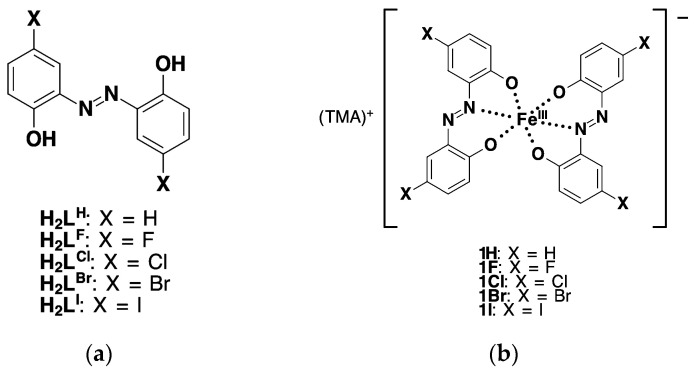
Structural formula of 2,2′-azobisphenol **H_2_L^X^** (**a**); (TMA)[Fe^III^(**L^X^**)_2_] **1X** [TMA = tetramethylammonium cation] (**b**).

2,2′-azobisphenol **H_2_L^H^** (Figure 1a) is known as an intense coloring and fluorometric reagent for various metal ions. Recently we discovered that an anionic homoleptic [Fe^III^(**L^H^**)_2_]^−^ complex **1H** (Figure 1b) [37] and its π-extended derivative [38], and a neutral heteroleptic [Fe^III^(**L^H^**)(qsal)] complex (qsal = *N*-(8-quinolyl)salicylaldiminate) [39] and its derivatives [35,40] showed gradual and abrupt SCO transitions, respectively. More recently, other groups also reported the SCO transitions in the [Fe(**L^H^**)_2_]^−^ complexes with other cations [41,42]. On the other hand, the substitution effect on the SCO behavior in the anionic [Fe(**L^H^**)_2_]^−^ complexes has not been reported to date. Therefore, we focused on the halogen substituent effect on SCO behavior in the anionic [Fe(**L^H^**)_2_]^−^ complexes. As dihalogen substituted ligands (Figure 1a), 5,5′-dichloro (**H_2_L^Cl^**) [43,44,45], 5,5′-dibromo (**H_2_L^Br^**) [43], 5,5′-diiodo (**H_2_L^I^**) [43] derivatives, and 4,4′-dibromo derivative [46] have been known so far. Although **H_2_L^Br^** and **H_2_L^I^** were synthesized by direct halogenation of **H_2_L^H^** [43], our attempt to synthesize **H_2_L^Br^** and **H_2_L^I^** using NBS [47] and NIS/*p*-TsOH [48] afforded a mixture of mono- to tri-substituted derivatives, making it difficult to isolate pure **H_2_L^Br^** and **H_2_L^I^**. Since SCO behavior is very sensitive to slight modifications in the crystal structure, introducing the number and position of substituents on the ligand must be number- and position-specific.

In this study, we investigated the synthetic routes for **H_2_L^X^** (Figure 1a, **X** = **F**, **Cl**, **Br**, **I**) and the crystal structures and magnetic properties of the anionic [Fe(**L^X^**)_2_]^−^ complexes **1X** (Figure 1b, **X** = **F**, **Cl**, **Br**, **I**). We could obtain pure **H_2_L^X^** by two synthetic routes and **1X** as nonsolvate crystals. Temperature variations in magnetic susceptibility and crystal structure for **1X** revealed that **1F**, **1Cl**, and **1Br** are isostructural, whose SCO transition temperatures are higher on increasing the size of halogen substituents. There are many short intermolecular contacts involved in C–H···π interactions, C–H···X hydrogen bonds, and X···X halogen bonds between the [Fe(**L^X^**)_2_]^−^ anions despite Coulomb repulsions. Halogen bonds between the same charged ions are rare and have been reported very recently in the literature [49,50]. Moreover, these intermolecular interactions can lead to the occurrence of a cooperative SCO transition in **1Cl** and **1Br** for the first time as the [Fe(**L^X^**)_2_]^−^ complexes. Furthermore, an SCO-induced rotational motion and relaxation to the original orientation of the ligands in **1Cl** and **1Br** were also observed, like the heteroleptic [Fe^III^(**L^H^**)(qsal)] derivative [40]. The presence of intermolecular interactions and the relaxation mechanism of ligand orientation are clarified from the temperature variation in intermolecular interaction energy using the topological analysis of electron density distributions.

## 2. Results and Discussion

### 2.1. Synthesis of Azobisphenols **H_2_L^X^**

**Scheme 1:**
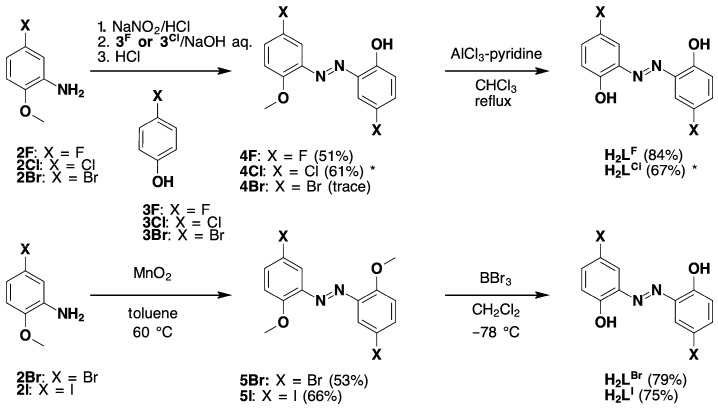
Synthesis of halogen-substituted azp ligands (**H_2_L^X^**). * The yields of **4Cl** and **H_2_L^Cl^** in the literature [43] were 41% and 56%, respectively.

The synthesis scheme is shown in Scheme 1. First, we investigate the reported synthesis method of the 5,5′-dichloro derivative **4Cl**, i.e., the diazo-coupling reaction of the diazonium cation obtained from *2*-methoxyaniline with phenolate anion followed by demethylation [45]. The reactivity of the 4-fluorophenolate anion toward electrophilic reagents is expected to be reduced due to the electron-withdrawing nature of fluorine. However, this is offset by the increased electrophilic reactivity of the 5-fluoro-*2*-methoxybenzene diazonium ion, and the difluoro derivative **4F** is obtained in a moderate yield. The subsequent demethylation reaction of **4F** with AlCl_3_-pyridine also proceeds, and the target 5,5′-difluoro azp derivative **H_2_L^F^** is obtained in a high yield. On the other hand, the diazo-coupling reaction between 5-bromo-*2*-methoxybenzene diazonium ion and 4-bromophenolate anion affords only trace amounts of the dibromo derivative **4Br**, even though the conditions are investigated. In fact, the diazo-coupling reactions with 4-bromo and 4-iodophenolate anions were not mentioned in the literature [51], probably because the electron transfer reaction between the diazonium and phenolate ions may be preferred [52].

Therefore, we investigate the reduction in nitrobenzene derivatives and oxidation of aniline derivatives for azobenzene synthesis. The reduction of 2-nitroanisole was examined using various reducing reagents, but all of them could afford a mixture of azoxybenzene, azobenzene, and aniline derivatives, and the desired azobenzene derivatives were not isolated. According to the literature [46], the oxidation reaction of 2-methoxyaniline derivatives (**2Br** and **2I**) with manganese dioxide can isolate the target azobenzene derivatives (**5Br** and **5I**) as a mixture of *trans*- and *cis*-isomers in a moderate yield. On the other hand, the demethylation reaction does not proceed under the same conditions as that of **4F**. Therefore, we examined the demethylation reaction of **5Br**. The demethylation with thiolate anion [53] gave a mixture of **H_2_L^Br^** and the undesired byproduct of debromination, whereas **H_2_L^Br^** and **H_2_L^I^** can be obtained using excess BBr_3_ in dilute conditions.

### 2.2. Synthesis of the Fe(III) Complexes **1X**

The anionic Fe(III) complexes with halogen-substituted ligands were prepared as tetramethylammonium (TMA) salts according to the literature procedure [37]. The compositions of the anionic [Fe(**L^X^**)_2_]^−^ complexes were confirmed by microanalyses and crystal analyses described below.

### 2.3. Magnetic Properties of the Fe(III) Complexes **1X**

**Figure 2:**
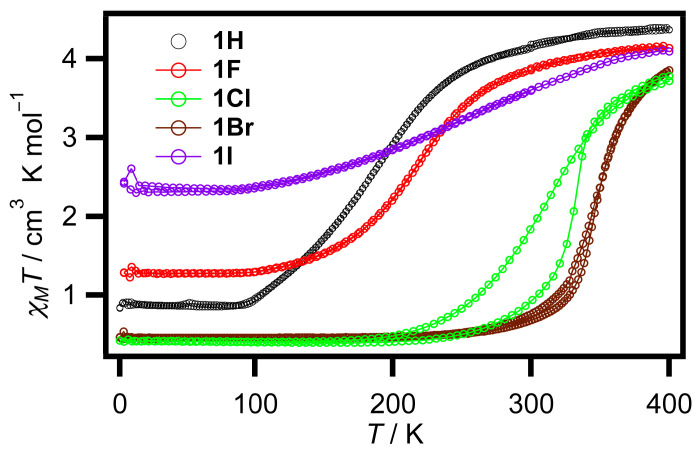
The *χ*_M_*T* vs. *T* product for the Fe(III) complexes **1X**.

The temperature variations in magnetic susceptibility for the complexes **1X** along with the parent complex **1H** [37] are shown in Figure 2.

The *χ*_M_*T* value for **1F** at 10 K is 1.28 cm^3^ K mol^−1^, indicative of a small amount of the HS fraction. On heating, the increase in the *χ*_M_*T* value starts at around 100 K. Then, the *χ*_M_*T* values gradually increase and reach 4.14 cm^3^ K mol^−1^ at 400 K, suggesting **1F** exhibits an incomplete gradual SCO conversion. No thermal hysteresis is observed for **1F**. The magnetic behavior of **1F** is similar to that of the parent non-substituted complex **1H**. When the SCO transition temperature (*T*_SCO_) is defined by the temperature given the maximum derivative of the *χ*_M_*T*–*T* curve, *T*_SCO_ for **1H** and **1F** are 194 and 220 K, respectively.

On the other hand, complex **1Cl** shows different magnetic behavior. The *χ*_M_*T* value for **1Cl** at 10 K was 0.41 cm^3^ K mol^−1^, indicating that **1Cl** is completely in the LS state of the Fe^III^ complex. On warming, the *χ*_M_*T* values for **1Cl** are almost constant, and then a slight increase is observed at around 220 K. Further increasing the temperature, an abrupt transition occurs around 315 K (*T*_SCO_ = 330 K). Above 340 K the slope of the *χ*_M_*T* vs. *T* plots turns gradual. The *χ*_M_*T* values reach 3.78 at 400 K. On consecutive cooling, the *χ*_M_*T* values decrease gradually down to 190 K (*T*_SCO_ = 314 K), whose process is different from the initial heating process. Further consecutive heating to 400 K, the *χ*_M_*T* values almost follow those in the preceding cooling process. This anomalous magnetic behavior is similar to that of the heteroleptic [Fe^III^(**L^H^**)(qsal)] derivative that showed a pedal-like rotational motion of the ligand in the HS state [40]. The changes in molecular structure associated with spin transitions in **1Cl** are discussed in the following section on crystal structure. After storing the measured sample at room temperature for one day, the *χ*_M_*T* values in the heating process reproduce those in the initial heating process. This behavior is also similar to that of the heteroleptic [Fe^III^(**L^H^**)(qsal)] derivative [40]. Figure S1 shows a graph of the time evolution of magnetic susceptibility at several temperatures. The relaxation of the *χ*_M_*T* values is observed at each temperature. However, it is not possible to fit the single relaxation process. The reason for this will also be discussed in the following section on crystal structure.

The *χ*_M_*T* value for **1Br** at 10 K is 0.45 cm^3^ K mol^−1^, indicating that **1Br** is completely in the LS state of the Fe^III^ complex. On warming, the *χ*_M_*T* values for **1Br** are almost constant, and then a slight increase is observed at around 280 K. Further increasing the temperature, an abrupt transition occurs around 320 K (*T*_SCO_ = 344 K). Above 380 K the slope of the *χ*_M_*T* vs. T plots turns gradual. The *χ*_M_*T* values reach 3.86 at 400 K. On consecutive cooling, the *χ*_M_*T* values follow those in the heating process, resulting in a very narrow thermal hysteresis between 300 and 340 K. Note that this is the first example of a homoleptic anionic [Fe(**L^X^**)_2_]^−^ complex that exhibits a cooperative SCO transition with a thermal hysteresis reversibly.

The *χ*_M_*T* value for **1I** at 10 K is 2.41 cm^3^ K mol^−1^, indicating that **1I** had a significant amount of the HS fraction. On warming, the *χ*_M_*T* values for **1I** gradually increase from 100 K to 400 K and reach 4.11 cm^3^ K mol^−1^ at 400 K. No thermal hysteresis is observed for **1I**.

### 2.4. Crystal Structures of the Fe(III) Complexes

#### 2.4.1. Description of Molecular Structure

To investigate the temperature and time variations in molecular and crystal structures on SCO transitions, the crystal structure analyses are performed on two crystals for **1F**, **1Cl**, and **1Br** and on three crystals for **1I** at various temperatures. All the crystallographic data are listed in Tables S1–S4.

**Figure 3:**
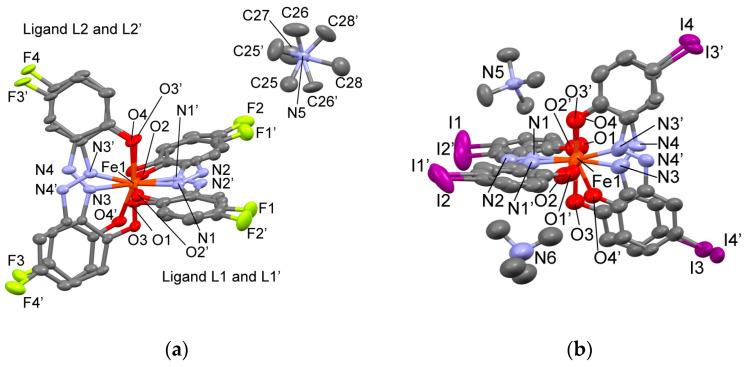
ORTEP drawings of 50% probability with selected atomic numbering for the asymmetric unit. (**a**) **1F** at 90 K; (**b**) **1I** at 90 K. See text for the occupancy of the TMA cation.

The crystal structures of **1F**, **1Cl**, and **1Br** are isostructural and belong to monoclinic *P*2_1_/*n*. The temperature and time variations in the crystal structures reveal no crystal structure phase transition for **1F**, **1Cl**, and **1Br** despite SCO transitions. Although the crystal structure of **1H** belonged to the same space group [37], the molecular arrangements of **1F**, **1Cl**, and **1Br** are different from that of **1H**. The asymmetric units of **1F**, **1Cl**, and **1Br** contain one TMA cation and one [Fe(**L^X^**)_2_]^−^ anion molecule. Figure 3a shows the asymmetric unit of **1F** at 90 K as a representative. The TMA cation in 1F exhibits a rotational disorder with the rotation axis along the C27-N5 bond, whereas those in **1Cl** and **1Br** are ordered at 90 K. The ligands in the [Fe(**L^X^**)_2_]^−^ anion also exhibit an orientation disorder. We hereafter refer to the major-oriented ligands with C1 to C12 and C13 to C24 atoms in **1Cl** and **1Br** at 90 K as ligands L1 and L2, respectively. The ligands with the opposite minor orientation and their atoms are labeled with a prime such as L1′ and C1′. The complex anion in **1Cl** and **1Br** at 90 K contains disordered ligands L1 and L1′ with L2. On the other hand, the ligands are all disordered in **1F**, whose major oriented ligands are ligands L1′ and L2.

The crystal structure of **1I** belongs to monoclinic *C*2/*c*, which was different from those of **1F**, 1Cl, and 1Br. The asymmetric unit of **1I** contains two half molecules of TMA cation and one [Fe(**L^I^**)_2_]^−^ anion molecule (Figure 3b). The N5 atom of one TMA cation is on the two-fold axis, and the occupancy of the C and H atoms in this cation is 50%. The N6 atom of the other TMA cation is close to the two-fold axis, and the occupancy of all the atoms of this cation is 50%. Therefore, the cations and ligands are all disordered in **1I**.

To clarify the relationship between the spinstate and coordination geometry of complexes, we compare coordination geometry parameters with those of **1H,** whose spin states were confirmed by the magnetic susceptibility and Mössbauer spectra [37]. The temperature dependence of the coordination geometry parameters bound to the major oriented ligands for **1F**, **1Cl**, **1Br**, **1I**, and **1H** are listed in Table 1 and Table S5. The bond lengths and distortion parameters Σ and Θ for **1F** at 90 K are very similar to those of **1H** at 90 K, which is in the LS state slightly mixed with the HS state. The Fe1-N3 bond lengths with ordered L2 in **1Cl** and **1Br** at 90 K are shorter than those of **1H** at 90 K. These are consistent with the magnetic susceptibility results that **1Cl** and **1Br** are completely in the LS state. The Fe1-N3 bond length, Σ, and Θ of **1I** at 90 K are intermediate values between 90 and 293 K of **1H**, suggesting the spin state of **1I** at 90 K is in the mixed spin state. The bond lengths, Σ, and Θ of **1F** at 293 K are almost the same as those of **1H** in the HS state, while the parameters of **1Cl** and **1Br** at 293 K are almost unchanged compared to 90 K, suggesting **1Cl** and **1Br** at 293 K are in the LS state. At 373 K, the parameters of **1F**, **1Cl**, **1Br**, and **1I** approached those of **1H** in the HS state. However, the Fe-N bond lengths of **1Cl** and **1Br** are about 0.05 Å shorter than those of **1H** in the HS state, which is consistent with the lower *χ*_M_*T* values in **1Cl** and **1Br** than that in **1H**.

#### 2.4.2. Temperature and Time Dependence of Disorder Ratios for **1F**, **1Cl**, and **1Br**

To investigate the reason for the anomalous magnetic behavior of **1Cl**, the ratios of TMA cation and ligands L1 and L2 with similar temperature and time sequence to magnetic susceptibility for the isostructural complexes **1F**, **1Cl**, and **1Br** are summarized in Table 2. Although the disorder of TMA cation is observed only in **1F** at 90 K, the disorder ratio of TMA cation is changed from 90 to 213 K, indicating the cation rotation occurs even below 213 K. This means the cation in **1F** exhibits a dynamic disorder. On the other hand, TMA cation in **1Cl** and **1Br** is ordered up to 293 K and then disordered at 373 K. This suggests that the cation rotation is coupled with SCO transitions. In fact, the disorder of the cation is not observed for both **1Cl** and **1Br** on cooling from 373 K to 90 K. This indicates that the anomalous magnetic behavior of **1Cl** must not arise from the cation rotation.

The ratios of both ligands L1 and L2 in **1Cl** and **1Br** are significantly changed from 293 to 373 K. These observations are reminiscent of a SCO-induced pedal-like rotational motion of ligand in the heteroleptic [Fe^III^(**L^H^**)(qsal)] derivative [40]. On the other hand, the variations in disorder ratios of ligands on cooling from 373 K to 90 K are different between **1Cl** and **1Br**. The ligand orientations of **1Cl** at 373 K are partly frozen, whereas those of **1Br** recover their original orientations. This suggests that the anomalous magnetic behavior of **1Cl** originates from a freezing of ligand orientational disorder. Furthermore, after the frozen crystal of **1Cl** is stored at room temperature for 7 and 38 days, the ratios of ligands reach constant ratios with 78% of L1 and 100% of L2. These values are in good agreement with those in other crystals at 90 K listed in Table S5. Therefore, the rotational motion of ligands in **1Cl** occurs even at room temperature, and the ligands can relax to more stable orientations. Note that the ratio of L1 is completely frozen, whereas that of L2 is partly frozen in **1Cl**. This is probably one of the reasons for the failure in fitting the relaxation of the *χ*_M_*T* values using a single relaxation process.

#### 2.4.3. Schematic Description of Molecular Arrangement in **1Br** and **1I** at 90 K

**Figure 4:**
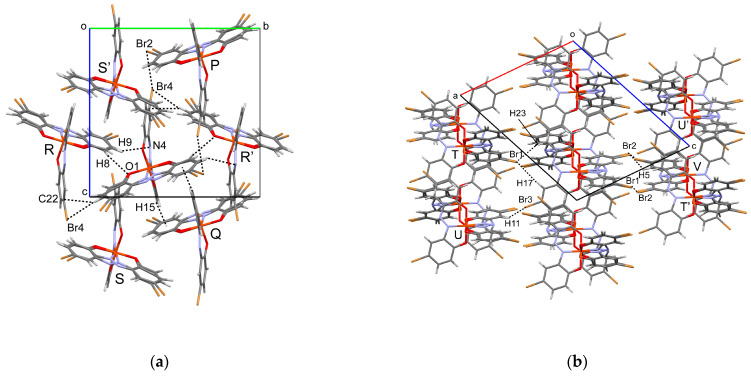
(**a**) Two-dimensional molecular network of the [Fe(**L^Br^**)_2_]^−^ anions in **1Br** along the *a* axis at 90 K. (**b**) Molecular arrangement between two-dimensional networks in **1Br** along the *b* axis at 90 K. Letters P–V with or without a prime are a label of the [Fe(**L^X^**)_2_]^−^ anion molecules with fractional coordinates described in the text and Table 3. Dot lines indicate selected intermolecular short contacts between the central reference [Fe(**L^X^**)_2_]^−^ anion molecule and the labeled one.

Stabilization of ligand orientation is considered to arise from intermolecular interactions because the [Fe(**L^X^**)_2_]^−^ anions with different ligand orientations have the same energy at the molecular level. Therefore, as a representative of the isostructural Fe complexes, the intermolecular arrangement of [Fe(**L^Br^**)_2_]^−^ anion molecules at 90 K is shown in Figure 4a,b. The molecular arrangement consists of characteristic interatomic short van der Waals contacts [54] between the [Fe(**L^Br^**)_2_]^−^ anions despite Coulomb repulsion. Table 3 and Table S6 in Supplementary Information list selected interatomic distances for each complex at various temperatures. There are short Br···Br contacts from a central reference [Fe(**L^Br^**)_2_]^−^ anion molecule with fractional coordinates (x, y, z) to molecule P with fractional coordinates (1 − x, 1 − y, 1 − z) and short C–H···π contacts to molecule Q with fractional coordinates (2 − x, 1 − y, 2 − z) (Figure 4a). These short contacts are alternately arranged along the *a* + *c* direction. Short C–H···O and C–H···N contacts, along with C–H···Br and C–H···C contacts, to molecules R with fractional coordinates (1.5 − x, −0.5 + y, 1.5 − z) and R′ with fractional coordinates (1.5 − x, 0.5 + y, 1.5 − z) are evident. The [Fe(**L^Br^**)_2_]^−^ anion molecules with these short contacts are arranged along the *b* axis. Furthermore, there are short C–H···O contacts to molecules S with fractional coordinates (0.5 + x, 0.5 − y, 0.5 + z) and S′ with fractional coordinates (−0.5 + x, 0.5 − y, −0.5 + z). Therefore, the [Fe(**L^Br^**)_2_]^−^ anion molecules form a two-dimensional (2D) network parallel to $\left[10\bar{1}\right]$ (Figure 4b). There are short C–H···Br contacts to molecules T with fractional coordinates (0.5 + x, 0.5 − y, −0.5 + z) and T′ with fractional coordinates (−0.5 + x, 0.5 − y, 0.5 + z), and U with fractional coordinates (1 + x, y, z) and U′ with fractional coordinates (−1 + x, y, z), and V with fractional coordinates (1 − x, 1 − y, 2 − z) between 2D networks.

**Figure 5:**
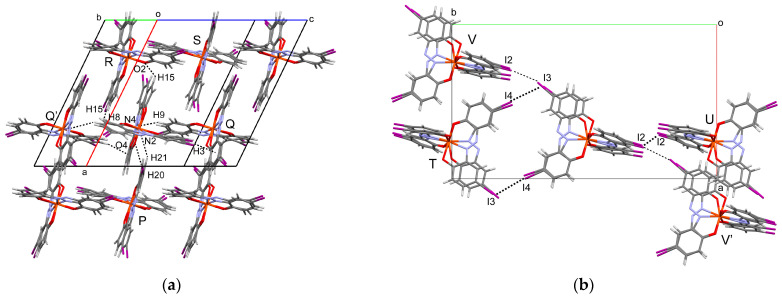
(**a**) Two-dimensional molecular network of [Fe(**L^I^**)_2_]^−^ anions in **1I** at 90 K. (**b**) Molecular arrangement between two-dimensional networks in **1I** along the *c* axis at 90 K. Letters P–V with or without a prime are a label of the [Fe(**L^I^**)_2_]^−^ anion molecules with fractional coordinates described in the text and Table 4. Dot lines indicate selected intermolecular short contacts between the central reference [Fe(**L^I^**)_2_]^−^ anion molecule and the labeled one.

The molecular arrangement of [Fe(**L^I^**)_2_]^−^ at 90 K is shown in Figure 5a,b. Table 4 and Table S6 in Supplementary Information list selected interatomic distances at various temperatures. **1I** also has a 2D molecular array of the [Fe(**L^I^**)_2_]^−^ anion molecules parallel to [020]. There are short C–H···N contacts to molecule P with fractional coordinates (2 − x, 1 − y, 1 − z) and short C–H···π contacts to molecules Q with fractional coordinates (x, 1 − y, −0.5 + z) and Q′ with fractional coordinates (x, 1 − y, 0.5 + z), resulting in the formation of a two-leg ladder-like arrangement along the *c* axis. However, there is no remarkable short contact between the two-leg ladder arrangements. The nearest π-plane distance between the two-leg ladder arrangements is 3.747 Å between the centroid···π-plane of molecule R with fractional coordinates (1 − x, y, 0.5 − z). The TMA cation of the N5 atom is located in the two-leg ladder arrangement, whereas that of the N6 atom is between the two-leg ladder arrangements. Different from C–H···X short contacts in isostructural complexes **1F**, **1Cl**, and **1Br**, there are short I···I contacts between 2D molecular arrangements in **1I**.

### 2.5. The Quantum Theory of Atom-in-Molecule (QTAIM) Analysis

#### 2.5.1. Comparison in Interaction Energy Between Isostructural Complexes **1F**, **1Cl**, and **1Br**

To gain an insight into short contacts and intermolecular interactions despite Coulomb repulsion between the [Fe(**L^X^**)_2_]^−^ anions, we calculate the electron density distribution of pairs of the central reference and the labeled [Fe(**L^X^**)_2_]^−^ anion molecules with L1 and L2 shown in Figure 4a,b from the crystal structures at 90 and 373 K by the density functional theory (DFT) method and perform the topological analysis using the quantum theory of atom-in-molecule (QTAIM) method [55]. The QTAIM analysis reveals that the (3,−1) critical points, namely bond critical points (BCPs), along the bond paths (BPs) between [Fe(**L^X^**)_2_]^−^ anion molecules, whose physical properties can indicate the nature of the interaction. All the properties of the BCPs between [Fe(**L^X^**)_2_]^−^ anion molecules for **1F**, **1Cl**, and **1Br** at 90 and 373 K are listed in Table S7. The intermolecular distance and interaction energy calculated from the density of all electrons (*ρ*(*r*)) at the BCP [56] are shown in Table 3. A significant value as intermolecular interaction is considered to be present when the interaction energy is less than −1.0 kJ mol^−1^.

The intermolecular short contacts in **1F** and **1Cl** at 90 K are found in similar locations as **1Br** described above. A notable difference is the absence of short F···F contacts to molecule P in **1F**. The QTAIM analysis gives the calculated interaction energies that support these observations. X···X halogen bonds are evident in **1Cl** and **1Br**, whereas there is no halogen bond in **1F** at 90 and 373 K. The location of the BP corresponding to the C–H···π interaction with molecule Q depends on the complex and temperature, but the interaction energy is almost the same. An additional C–H···O hydrogen bond with molecule Q is also found in **1F**. Other intermolecular interactions, such as C–H···N and C–H···O hydrogen bonds to molecules R and S, and C–H···X hydrogen bonds to molecules T, U, and V, are found with comparable interaction energies. Moreover, the BCPs along the BPs other than short contacts mentioned above reveal that weak halogen and hydrogen bonds exist with slightly longer C–H···X and X···X distances than the sum of the van der Waals radii.

At 373 K, the interactions with molecule S are notably weakened for all complexes. On the other hand, the C–H···X hydrogen bonds to molecules T and V between 2D networks are also weakened only for **1F**. Therefore, the cooperative nature of SCO in **1Cl** and **1Br** may be related to the X···X halogen bonds within 2D networks and these C–H···X hydrogen bonds between 2D networks. There is no significant difference in interaction energy between **1Cl** and **1Br**. Thus, we cannot conclude why only **1Cl** shows frozen ligand orientations from the point of view of intermolecular interaction. One possible explanation is the relationship between the SCO transition temperature and the activation energy of the rotational motion of the ligand. Since **1Cl** has a lower SCO transition temperature than **1Br**, the energy in the SCO transition temperature of **1Cl** cannot exceed the activation energy of the ligand motion in **1Cl**, but that of **1Br** can exceed it in **1Br**. Thus, the ligand orientations can be frozen in **1Cl**.

#### 2.5.2. Comparison Between Major and Minor Ligand Orientations in **1Br** at 90 K

The ligand orientations can be considered to depend on the difference in the lattice energy of each ligand orientation. The intermolecular interactions between the TMA cations and [Fe(**L^X^**)_2_]^−^ anions are mainly long-range electrostatic interactions, and the difference in electrostatic energy between the ligand orientations may be negligible. In addition, intermolecular interactions far from the nearest neighbor [Fe(**L^X^**)_2_]^−^ anions are mainly short-range dispersion interactions, and there must be no difference between ligand orientations. Therefore, we should compare the intermolecular interaction energies between the nearest neighbor [Fe(**L^X^**)_2_]^−^ anions for each ligand orientation. Since there are only two ligand orientation complex anions in the low-temperature phase of **1Br**, we will compare the contributions of the nearest neighbor interaction energies to the lattice energy between the major orientation complex anion with 86.9% of L1 and 100% of L2 and the minor orientation complex anion with 13.1% of L1′ and 100% of L2. All the properties of the BCPs between [Fe(**L^Br^**)_2_]^−^ anion molecules with minor orientation at 90 K are listed in Table S8. The calculated energies are shown in Table 5.

The contribution of the nearest neighbor interactions to the lattice energy of one [Fe(**L^Br^**)_2_]^−^ anion must consider the number of equivalent molecular interactions, that is, the number of equivalent intermolecular interactions within the same molecular pair (*N*_equiv_) and the number of different molecular pairs with an equivalent intermolecular interaction (*N*_pair_). The contribution of the nearest neighbor interactions to the total intermolecular interactions must be divided by two due to double counting the same intermolecular interactions in the crystal. Therefore, the contribution of the nearest neighbor interaction energy (*E*_NN_) can be calculated using the following Equation (1).

(1) $$E_{\mathrm{NN}}=\sum_{n}\frac{E_{n}\times N_{\mathrm{equiv}}\times N_{\mathrm{pair}}}{2}$$

The value of *E*_NN_ for the [Fe(**L^Br^**)_2_]^−^ anion with the major ligand orientation is −69.0 kJ mol^−1^, whereas that with the minor ligand orientation is −64.4 kJ mol^−1^. This means the [Fe(**L^Br^**)_2_]^−^ anion with the major orientation is more stabilized than that with the minor orientation. This may be one of the reasons that the ratio of ligand orientations is recovered on SCO from the HS to LS states in **1Br**.

#### 2.5.3. Comparison in Interaction Energy Between 90 and 373 K in **1I**

To estimate the intermolecular interaction energy for short contacts in **1I**, we perform the topological analysis for the electron density distribution of pairs of the central reference and the labeled [Fe(**L^I^**)_2_]^−^ anion molecules with L1 and L2 shown in Figure 5a,b at 90 and 373 K using the QTAIM method [55]. The intermolecular distance and interaction energy at the BCPs in **1I** are listed in Table 4.

As mentioned in the molecular arrangement in **1I**, the two-leg ladder arrangement of the [Fe(**L^X^**)_2_]^−^ anion consists of C–H···N hydrogen bonds to molecule P and C–H···π interactions and C–H···N hydrogen bonds to molecules Q and Q′. There are weak intermolecular interactions between molecules R and S in the two-leg ladder arrangements at 90 K. On the other hand, there are moderate interaction energies of I···I halogen bonds to molecules T and U between 2D layers. Therefore, from the point of intermolecular interactions, a two-dimensional interaction network forms parallel to the *bc* plane in **1I**.

## 3. Materials and Methods

All the chemicals were purchased and used without further purification. 1,2-bis(5-chloro-2-hydroxyphenyl)diazene (**H_2_L^Cl^**) was prepared according to the literature [45]. Improved yields concerning the synthesis of **H_2_L^Cl^** are shown in Scheme 1. ^1^H NMR spectra were recorded on a Bruker Avance 400 spectrometer. Elemental analyses were performed on a Yanaco CHN corder MT-5 elemental analyzer. Variable temperature direct current magnetic susceptibilities of polycrystalline samples were measured on a Quantum Design MPMS-XL magnetometer under a field of 0.5 T in the temperature range of 2 or 5 to 400 K. The magnetic susceptibilities were corrected for diamagnetic contributions estimated by Pascal constants [57].

### 3.1. Synthesis of Ligands

#### 3.1.1. 1-(5-Fluoro-2-hydroxyphenyl)-2-(5-fluoro-2-methoxyphenyl)diazene (**4F**)

A suspension of 5-fluoro-2-methoxyaniline **2F** (0.83 mL, 7.09 mmol) in 2.0 mL of water and 1.8 mL of conc. HCl was stirred and cooled to 0 °C. To the suspension was added dropwise a solution of NaNO_2_ (538 mg, 7.80 mmol) in 1.2 mL of water. The mixture turned out to be a reddish-brown solution and kept below 5 °C. After confirming the existence of HNO_2_ using a KI-starch paper, the solution was quickly transferred to a solution of 4-fluorophenol **3F** (794 mg, 7.09 mmol) in 5 mL of an aqueous solution of NaOH (453 mg, 11.3 mmol). The reaction mixture was stirred for 2 h below 5 °C and then warmed to room temperature. The precipitate was filtered. To this suspension was added 2 mL of 3.5% HCl and stirred. The precipitate was filtered and dried in vacuo. Recrystallization from ethyl acetate gave **4F** (954 mg, 51%) as reddish-purple needles.

^1^H NMR (400 MHz, CDCl_3_) δ 13.06 (s, 1 H), 7.64 (dd, *J*_H-F_ = 8.7 Hz, *J*_H-H_ = 3.1 Hz, 1 H), 7.59 (dd, *J*_H-F_ = 9.0 Hz, *J*_H-H_ = 3.1 Hz, 1 H), 7.20 (ddd, *J*_H-F_ = 7.5 Hz, *J*_H-H_ = 9.1, 3.1 Hz, 1 H), 7.09 (ddd, *J*_H-F_ = 7.8 Hz, *J*_H-H_ = 9.1, 3.1 Hz, 1 H), 7.04 (dd, *J*_H-F_ = 4.4 Hz, *J*_H-H_ = 9.1 Hz, 1 H), 6.99 (dd, *J*_H-F_ = 4.8 Hz, *J*_H-H_ = 9.1 Hz, 1 H), 4.00 (s, 3 H) ppm. Anal. Calcd. for C_13_H_10_F_2_N_2_O_2_: C, 59.09; H, 3.81; N, 10.60%. Found: C, 59.10; H, 3.78; N, 10.54%.

#### 3.1.2. 1,2-Bis(5-fluoro-2-hydroxyphenyl)diazene (**H_2_L^F^**)

To a stirred solution of **4F** (1.32 g, 5.00 mmol) in 100 mL of chloroform was added, portionwise, a finely powdered AlCl_3_ (2.67 g, 20.0 mmol). The resulting red suspension was warmed to 30 °C, and then 10.5 mL of pyridine was added dropwise to this suspension. The mixture was heated to reflux for 90 min and then cooled to room temperature. After reducing the volume of the mixture to about a quarter of its original volume using an evaporator, it was poured into 20 mL of methanol and then acidified by adding 20 mL of 36% HCl and 20 mL of water. The mixture was heated at 80 °C for two hours. The resulting precipitate was filtered and dried in vacuo. Recrystallization from ethyl acetate gave **H_2_L^F^** as orange needle crystals (1.03 g, 84%).

^1^H NMR (400 MHz, CDCl_3_) δ 11.76 (s, 2 H), 7.45 (dd, *J*_H-F_ = 8.4 Hz, *J*_H-H_ = 3.0 Hz, 2 H), 7.14 (ddd, *J*_H-F_ = 7.6 Hz, *J*_H-H_ = 9.1, 3.0 Hz, 2 H), 7.02 (dd, *J*_H-F_ = 4.8 Hz, *J*_H-H_ = 9.2 Hz, 2 H) ppm. Anal. Calcd. for C_12_H_8_F_2_N_2_O_2_: C, 57.61; H, 3.22; N, 11.20%. Found: C, 57.71; H, 3.16; N, 11.24%.

#### 3.1.3. 1,2-Bis(5-bromo-2-methoxyphenyl)diazene (**5Br**)

A mixture of 5-Bromo-2-methoxyaniline **2Br** (4.00 g, 19.8 mmol), MnO_2_ (6.88 g, 79.2 mmol), and toluene (80 mL) was heated to 70 °C for 24 h. After cooling, the precipitate was filtered and washed with hot chlorobenzene three times. The hot filtrate was cooled, and the precipitate was filtered to afford **5Br** (2.10 g, 53%) as orange needle crystals.

^1^H NMR (400 MHz, CDCl_3_) δ (*trans*) 7.75 (d, *J* = 2.5 Hz, 2 H), 7.53 (dd, *J* = 8.8, 2.5 Hz, 2 H), 6.97 (d, *J* = 8.9 Hz, 2 H), 4.02 (s, 6 H); (*cis*) 7.21 (dd, *J* = 8.8, 2.5 Hz, 2 H), 6.84 (d, *J* = 2.4 Hz, 2 H), 6.66 (d, *J* = 8.8 Hz, 2 H), 3.72 (s, 6 H) ppm. Anal. Calcd. for C_14_H_12_Br_2_N_2_O_2_: C, 42.03; H, 3.02; N, 7.00%. Found: C, 42.05; H, 2.97; N, 6.91%.

#### 3.1.4. 1,2-Bis(5-bromo-2-hydroxyphenyl)diazene (**H_2_L^Br^**)

A solution of **5Br** (500 mg, 1.25 mmol) in 150 mL of CH_2_Cl_2_ was cooled to −94 °C. To the solution was added 1 M CH_2_Cl_2_ solution of BBr_3_ (31 mL, 31.2 mmol). The reaction mixture was warmed and stirred at room temperature for one night. After cooling to 0 °C, MeOH was carefully added to quench the reaction. The precipitate was collected and washed with MeOH to afford **H_2_L^Br^** (364 mg, 79%) as a yellow powder.

^1^H NMR (400 MHz, CDCl_3_) δ 11.91 (s, 2 H), 7.87 (d, *J* = 2.4 Hz, 2 H), 7.46 (dd, *J* = 8.9, 2.4 Hz, 2 H), 6.97 (d, *J* = 8.9 Hz, 2 H) ppm. Anal. Calcd. for C_12_H_8_Br_2_N_2_O_2_: C, 38.74; H, 2.17; N, 7.53%. Found: C, 38.66; H, 2.13; N, 7.51%.

#### 3.1.5. 1,2-Bis(5-iodo-2-methoxyphenyl)diazene (**5I**)

A mixture of 5-iodo-2-methoxyaniline **2I** (5.00 g, 20.1 mmol), MnO_2_ (6.98 g, 80.3 mmol), and toluene (80 mL) was heated to 70 °C for 72 h. After cooling, the precipitate was filtered and washed with hot chlorobenzene several times. After cooling the filtrate, the precipitate was filtered to afford **5I** (3.29 g, 66%) as an orange powder.

^1^H NMR (400 MHz, CDCl_3_) δ (*trans*) 7.86 (d, *J* = 2.3 Hz, 2 H), 7.71 (dd, *J* = 8.7, 2.3 Hz, 2 H), 6.85 (d, *J* = 8.8 Hz, 2 H), 4.01 (s, 6 H); (*cis*) 7.38 (dd, *J* = 8.7, 2.1 Hz, 2 H), 7.01 (d, *J* = 2.2 Hz, 2 H), 6.54 (d, *J* = 8.7 Hz, 2 H), 3.70 (s, 6 H) ppm. Anal. Calcd. for C_14_H_12_I_2_N_2_O_2_: C, 34.03; H, 2.45; N, 5.67%. Found: C, 34.11; H, 2.41; N, 5.61%.

#### 3.1.6. 1,2-Bis(5-iodo-2-hydroxyphenyl)diazene (**H_2_L^I^**)

A solution of **5I** (433 mg, 0.875 mmol) in 430 mL of CH_2_Cl_2_ was cooled to −94 °C. To the solution was added 1 M CH_2_Cl_2_ solution of BBr_3_ (88 mL, 88.0 mmol). The reaction mixture was warmed and stirred at room temperature for one night. After cooling to 0 °C, MeOH was carefully added to quench the reaction. The precipitate was collected and washed with MeOH to afford **H_2_L^I^** (306 mg, 75%) as an orange powder.

^1^H NMR (400 MHz, CDCl_3_) δ 11.93 (s, 2 H), 8.04 (d, *J* = 2.2 Hz, 2 H), 7.62 (dd, *J* = 8.9, 2.2 Hz, 2 H), 6.85 (d, *J* = 8.8 Hz, 2 H) ppm. Anal. Calcd. for C_12_H_8_I_2_N_2_O_2_: C, 30.93; H, 1.73; N, 6.01%. Found: C, 30.75; H, 1.67; N, 5.95%.

### 3.2. Synthesis Procedure of the [Fe^III^(**L^X^**)_2_] Complexes (**1X**)

To a suspension of **H_2_L^X^** (2.00 mmol) in 18 mL of methanol was added dropwise a 28% methanol solution of sodium methoxide (0.89 mL, 4.40 mmol) diluted with 4 mL of methanol. The suspension was heated to 60 °C and stirred to turn into a dark solution. To this solution was added a solution of FeCl_3_ (162 mg, 1.00 mmol) in 9 mL of methanol. The solution was heated to reflux for 100 min and then cooled to room temperature. To the solution was added tetramethylammonium bromide (385 mg, 2.5 mmol) in 20 mL of methanol, and then the solution was stirred for 30 min. After evaporating the solution to a few mL, water was added. The precipitate was filtered and washed with water.

#### 3.2.1. TMA[Fe^III^(**L^F^**)_2_] (**1F**)

**1F** was synthesized by using **H_2_L^F^** (500 mg, 2.00 mmol). Recrystallization of the crude salt from acetone-diethyl ether gave **1F** (485 mg, 77%) as black platelets. Anal. Calcd. for C_28_H_24_F_4_FeN_5_O_4_: C, 53.69; H, 3.86; N, 11.18%. Found: C, 53.78; H, 3.90; N, 11.36%.

#### 3.2.2. TMA[Fe^III^(**L^Cl^**)_2_] (**1Cl**)

**1Cl** was synthesized by using **H_2_L^Cl^** (425 mg, 1.50 mmol). Recrystallization of the crude salt from acetone-diethyl ether gave **1Cl** (130 mg, 25%) as black platelets. Anal. Calcd. for C_28_H_24_Cl_4_FeN_5_O_4_: C, 48.59; H, 3.49; N, 10.12%. Found: C, 48.52; H, 3.50; N, 10.31%.

#### 3.2.3. TMA[Fe^III^(**L^Br^**)_2_] (**1Br**)

**1Br** was synthesized by using **H_2_L^Br^** (300 mg, 0.806 mmol). Recrystallization of the crude salt from acetone-diethyl ether gave **1Br** (160 mg, 46%) as black platelets. Anal. Calcd. For C_28_H_24_Br_4_FeN_5_O_4_: C, 38.66; H, 2.78; N, 8.05%. Found: C, 38.57; H, 2.75; N, 8.00%.

#### 3.2.4. TMA[Fe^III^(**L^I^**)_2_] (**1I**)

**1I** was synthesized by using **H_2_L^I^** (160 mg, 0.343 mmol). Recrystallization of the crude salt from methanol-diethyl ether gave **1I** (127 mg, 70%) as black needles. Anal. Calcd. for C_28_H_24_FeI_4_N_5_O_4_: C, 31.79; H, 2.29; N, 6.62%. Found: C, 31.54; H, 2.28; N, 6.43%.

### 3.3. Single Crystal X-Ray Diffractions

A crystal was mounted on a roll of 15 μm thick polyimide film by using the Araldite^TM^ adhesive. A Nihon Thermal Engineering nitrogen gas flow temperature controller was used for the temperature variable measurements. All data were collected on a Bruker APEX II CCD area detector with monochromated Mo-Kα radiation generated by a Bruker Turbo X-ray Source coupled with Helios multilayer optics. All data collections were performed using the APEX2 crystallographic software package (Bruker AXS, Billerica, MA, USA). The data were collected to a maximum 2*θ* value of 55.0°. A total of 720 oscillation images were collected. The APEX3 crystallographic software package (Bruker AXS) was used to determine the unit cell parameters. Data were integrated by using SAINT. Numerical absorption correction was applied by using SADABS. The structures at all temperatures were solved by direct methods and refined by full-matrix least-squares methods based on *F*^2^ by using the SHELXTL program. All non-hydrogen atoms were refined anisotropically. Hydrogen atoms were generated by calculation and refined using the riding model. Since the structures of **1I** at 293 and 373 K were not converged due to heavy disorder of one TMA cation, those were determined applying the SQUEEZE procedure [58] to the TMA cation. CCDC 2391887–2391906 contains the supplementary crystallographic data for this paper. These data can be obtained free of charge via https://www.ccdc.cam.ac.uk/structures/? (accessed on 16 November 2024) (or from the CCDC, 12 Union Road, Cambridge CB2 1EZ, UK; Fax: +44 1223 336033; E-mail: deposit@ccdc.cam.ac.uk).

### 3.4. Density Functional Theory (DFT) Calculations

The atomic coordinates were taken from the experimental X-ray atomic coordinates for **1X** at 90 and 373 K. The [Fe(**L^X^**)_2_]^−^ anions of **1F**, **1Cl**, and **1Br** at 90 and 373 K were designated the LS and HS states, respectively. The [Fe(**L^I^**)_2_]^−^ anions at 90 and 373 K were defined as the HS state. Hydrogen atoms were optimized at the B3LYP level [59,60] using the Gaussian 16 program package [61]. The 6-311+G(d,p) basis sets were used for H [62], C, N, F [62,63], and Cl [64,65,66] atoms. The Wachters–Hay [67,68] basis sets were used for the Fe atom, and the LanL2DZ [69] basis set was used for the Br and I atoms. For electron density distributions, single-point calculations based on supramolecular dimers generated by the symmetry operations of the hydrogen-optimized atomic geometries of the [Fe(**L^X^**)_2_]^−^ anions were performed at the M06 level [70] using the Gaussian 16 program package [61]. Douglas–Kroll–Hess second-order scalar relativistic core Hamiltonian calculations were carried out using the DZP-DKH basis sets for the I atom [71] and all other atoms [72]. The topological analysis based on the QTAIM method [55] of the electron density distributions was performed using the Multiwfn program [73]. Since the interaction energy estimated using the equation for charged dimers gave larger values at all BCPs with or without short contacts, the interaction energy was calculated from the density of all electrons at each BCP using the equation for neutral dimers in the literature [56].

## 4. Conclusions

To investigate the halogen substitution effect on anionic spin crossover (SCO) complexes, we synthesized azobisphenolate ligands with 5,5′-dihalogen substituents and their anionic Fe^III^ complexes **1F**, **1Cl**, **1Br**, and **1I**. The temperature dependence of magnetic susceptibility, crystal structure, and topological analysis of the electron density distributions for isostructural complexes **1F**, **1Cl**, and **1Br** revealed that the existence of X···X halogen bonds, C–H···X, C–H···N, and C–H···O hydrogen bonds, and C–H···π interactions between the [Fe(**L^X^**)_2_]^−^ anions with the rotational motion of the cation and ligands leads to gradual, irreversible, and reversible cooperative SCO transitions for **1F**, **1Cl**, and **1Br**, respectively. This indicates that the SCO transitions in the isostructural [Fe(**L^X^**)_2_]^−^ anion complexes originate from complex mechanisms due to not only inductive effects of halogen substituents but also molecular motions and intermolecular interactions. Therefore, we can demonstrate that the temperature variation in the crystal structure and intermolecular interaction energy provide important insights into the transition mechanism of SCO crystals. Moreover, halogen-substituted ligands are useful as synthetic intermediates and are expected to lead to the synthesis of various functional ligand derivatives. The synthesis of new functional azobisphenolate ligands is now in progress.

## Figures and Tables

**Table 1:** Coordination bond length (Å) and distortion parameters Σ and Θ (°) of **1F**, **1Cl**, **1Br**, **1I**, and **1H** at selected temperatures.

Complex	1F ^a^			1Cl			1H [35]	
Temp./K	90	293	373	90	293	373	90	293
Fe1-O1	1.909 (4)	1.961 (8)	1.961 (7)	1.941 (2)	1.930 (3)	1.981 (8)	1.9233 (15)	1.975 (2)
Fe1-O2	1.908 (4)	1.932 (9)	1.954 (7)	1.857 (2)	1.886 (3)	1.920 (9)	1.8593 (16)	1.947 (2)
Fe1-N1	1.945 (4)	2.145 (5)	2.164 (3)	1.9189 (19)	1.960 (2)	2.128 (3)	1.9523 (16)	2.166 (2)
Fe1-O3	1.912 (3)	1.959 (6)	1.968 (5)	1.9267 (14)	1.9273 (18)	1.967 (3)	1.9217 (13)	1.9836 (19)
Fe1-O4	1.913 (4)	1.941 (6)	1.951 (5)	1.8735 (15)	1.8878 (18)	1.940 (3)	1.8898 (13)	1.942 (2)
Fe1-N3	1.927 (3)	2.123 (5)	2.143 (3)	1.9103 (14)	1.9512 (18)	2.107 (2)	1.9220 (14)	2.1537 (19)
Σ ^c^	42.4 (8)	89.8 (13)	98.5 (10)	43.1 (5)	51.5 (9)	76.4 (11)	39.4 (3)	89.6 (3)
Θ ^d^	48.8 (9)	135.7 (14)	157.5 (11)	54.5 (5)	64.5 (10)	125.7 (12)	53.2 (3)	169.6 (4)
	**1Br**			**1I**				
Temp./K	90	293 ^b^	373	90	373 ^b^			
Fe1-O1	1.935 (2)	1.947 (5)	1.961 (5)	2.021 (9)	2.010 (8)			
Fe1-O2	1.857 (2)	1.867 (5)	1.924 (6)	1.846 (10)	1.919 (9)			
Fe1-N1	1.9050 (19)	1.915 (4)	2.075 (5)	2.033 (7)	2.139 (4)			
Fe1-O3	1.9197 (15)	1.932 (4)	1.960 (6)	2.021 (9)	2.010 (9)			
Fe1-O4	1.8833 (15)	1.891 (3)	1.925 (6)	1.846 (10)	1.917 (9)			
Fe1-N3	1.8985 (17)	1.915 (4)	2.074 (4)	2.031 (7)	2.139 (4)			
Σ ^c^	27.1 (5)	31.9 (7)	71.8 (18)	76 (2)	100.2 (17)			
Θ ^d^	36.9 (5)	42.3 (7)	111.3 (18)	83 (3)	142.3 (18)			

^a^ Coordination geometry with ligands L1′ and L2. ^b^ Another crystal different from the crystal at other temperatures. ^c^ The sum of the absolute differences of 12 bite angles from 90°. ^d^ The sum of the absolute differences of 24 angles of 8 triangular surfaces of a coordination octahedron from 60°.

**Table 2:** Temperature and time sequence dependence of disorder ratios of cation and ligands in **1F**, **1Cl**, and **1Br**.

Complex		1F				1Cl				1Br			
Analysis Temp.	Storage Time at rt	Seq. No. ^a^	L1	L2	Cation	Seq. No. ^a^	L1	L2	Cation	Seq. No. ^a^	L1	L2	Cation
90 K	–	1	28.1%	74.0%	71.8%	1	50.7%	100%	100%	1	86.9%	100%	100%
213 K	–	2	28.6%	69.9%	50.8%								
293 K	–	3	29.9%	71.5%	41.9%	2	49.0%	100%	100%		85.4% ^b^	100% ^b^	100% ^b^
373 K	–	4	36.2%	67.8%	46.5%	3	42.1%	79.4%	58.1%	2	55.4%	81.7%	51.2%
90 K	–					4	42.2%	92.3%	100%				
90 K	1 day									3	86.9%	100%	100%
90 K	7 days					5	71.5%	100%	100%				
90 K	38 days					6	78.1%	100%	100%				

^a^ Measurement sequence number. ^b^ Another crystal different from the crystal at other temperatures.

**Table 3:** Intermolecular distance and interaction energy between the reference and labeled [Fe(**L^X^**)_2_]^−^ anion molecules from the density of all electrons at the BCPs for **1F**, **1Cl**, and **1Br** at 90 and 373 K. The numbers in square brackets represent the sum of van der Waals radii [54].

Contact or Bond Path	Intermolecular Distance (Å)						Interaction Energy (kJ mol^−1^)					
Complex	1F		1Cl		1Br		1F		1Cl		1Br	
Temp. (K)	90	373	90	373	90	373	90	373	90	373	90	373
to P (1 − x, 1 − y, 1 − z)												
X2···X4	3.032 (11)	3.06 (2)	3.3455 (14)	3.373 (7)	3.5088 (5)	3.534 (7)	−0.5	−0.3	−5.4	−4.9	−6.3	−6.0
	[2.94]		[3.50]		[3.70]							
to Q (2 − x, 1 − y, 2 − z)												
H15···centroid (C7–C12)	2.5945	2.6917	2.4614	2.6825	2.4615	2.6770						
C15···centroid (C7–C12)	3.520 (14)	3.55 (2)	3.360 (3)	3.492 (15)	3.357 (4)	3.48 (2)						
H15···C7	2.6602	2.9334	2.8543	3.1998	2.9107	3.2866	−5.9	–	–	–	–	–
H15···C8	2.7242	2.9433	2.9408	3.0986	2.9368	3.3233	–	−2.4	–	–	–	–
H15···C10	3.2131	3.1066	2.7526	2.7908	2.7327	2.6620	–	–	−5.5	−5.0	–	−7.4
H15···C10-C11							–	–	–	–	−6.1	–
H14···C7	3.0840	2.8946	3.0785	3.1296	3.0963	3.1005	–	–	–	>0	−0.0	–
H14···C8	3.3794	3.2741	3.3040	3.2975	3.2869	3.1662	–	–	–	–	–	-0.4
	[2.90]											
**Contact or Bond Path**	**Intermolecular Distance (Å)**						**Interaction Energy (kJ mol^−1^)**					
Complex	**1F**		**1Cl**		**1Br**		**1F**		**1Cl**		**1Br**	
Temp. (K)	90	373	90	373	90	373	90	373	90	373	90	373
to Q (2 − x, 1 − y, 2 − z)												
H14···O2	2.6713	2.6135	2.9450	3.1216	3.0635	3.1427	−3.5	−4.8	−0.6	–	–	–
H14···O3	2.8543	3.0646	3.0544	3.6018	3.2414	3.7105	−1.6	–	–	–	–	–
	[2.72]											
X2···X3	4.851 (11)	4.96 (2)	4.1571 (15)	4.320 (8)	4.1230 (5)	4.304 (8)	–	–	>0	>0	−0.7	>0
	[2.94]		[3.50]		[3.70]							
to R (1.5 − x, −0.5 + y, 1.5 − z)												
N4···H9	2.6694	2.5993	2.6458	2.5469	2.6371	2.6960	−5.2	−6.0	−5.2	−6.2	−5.1	−4.6
	[2.75]											
O1···H8	2.7085	2.6326	2.4720	2.5645	2.4892	2.5238	−3.9	−4.8	−7.1	−5.5	−6.8	−5.8
	[2.72]											
H3···X4	3.2084	3.4518	3.0082	3.5009	3.0382	3.4622	–	–	–	–	−4.3	–
	[2.67]		[2.95]		[3.05]							
H3···C22	2.9022	2.8647	2.8315	2.9380	2.8377	2.8304	−2.4	−4.0	−3.4	−3.1	−4.1	−5.0
	[2.90]											
to S (0.5 + x, 0.5 − y, 0.5 + z)												
O1···H21	2.5936	2.9647	2.5278	2.9213	2.4916	2.8781	−5.1	−0.7	−6.7	−1.1	−7.6	−1.8
	[2.72]											
C2···H20	2.7542	3.0593	2.9676	2.9895	3.1384	3.2637	−3.3	−0.4	−1.4	−1.1	−0.1	>0
	[2.90]											
C14···C21	3.585 (16)	3.86 (3)	3.568 (3)	3.628 (16)	3.514 (3)	3.79 (2)	−0.9	–	−1.3	−0.6	−1.6	>0
C15···C22	3.500 (13)	3.66 (2)	3.615 (3)	3.655 (12)	3.653 (3)	3.81 (3)	–	–	–	−0.4	–	–
C16···C22	3.300 (10)	3.48 (2)	3.452 (2)	3.672 (16)	3.575 (3)	3.83 (3)	−2.3	−0.7	−1.7	–	–	–
	[3.40]											
C16···X4	3.210 (8)	3.336 (17)	3.542 (2)	3.701 (11)	3.668 (2)	3.83 (2)	−2.0	−0.6	–	−1.0	–	−1.3
C17···X4	3.245 (7)	3.463 (13)	3.491 (5)	3.715 (8)	3.605 (2)	3.839 (11)	–	–	−2.7	–	−2.9	–
	[3.17]	[3.45]	[3.55]									
X3···X4	3.250 (7)	3.343 (15)	3.6498 (9)	3.813 (6)	3.8714 (4)	4.096 (6)	–	–	−2.4	−1.0	−2.8	−0.9
	[2.94]	[3.50]	[3.70]									
to T (0.5 + x, 0.5 − y, −0.5 + z)												
H23···X1	2.8439	2.9519	2.9087	3.0831	3.0390	3.4138	−0.7	>0	−4.2	−2.3	−4.2	−0.8
H17···X1	2.6203	2.7648	3.0525	3.1530	3.2614	3.0868	−2.7	−1.1	−1.8	−1.2	−1.2	−3.1
	[2.67]		[2.95]		[3.05]							
to U (1 + x, y, z)												
X3···H11	2.4638	2.5917	2.8406	2.9187	2.8784	2.9790	−5.9	−3.7	−4.8	−3.8	−6.4	−4.7
	[2.67]		[2.95]		[3.05]							
to V (1 − x, 1 − y, 2 − z)												
X2···H5	2.6888	2.7849	2.7572	2.8543	2.8570	3.0876	−2.2	−0.6	−6.1	−4.1	−6.1	−2.9
	[2.67]		[2.95]		[3.05]							
X1···X2	4.224 (15)	4.45 (3)	3.770 (3)	4.292 (11)	3.8522 (16)	4.190 (11)	–	–	−1.2	–	−2.7	−0.0
	[2.94]		[3.50]		[3.70]							

**Table 4:** Intermolecular distance and interaction energy between the reference and labeled [Fe(**L^I^**)_2_]^−^ anion molecules from the density of all electrons at the BCPs for **1I** at 90 and 373 K. The numbers in square brackets represent the sum of van der Waals radii [54].

Contact or Bond Path	Intermolecular Distance (Å)		Interaction Energy (kJ mol^−1^)		Contact or Bond Path	Intermolecular Distance (Å)		Interaction Energy (kJ mol^−1^)	
Temp. (K)	90	373	90	373		90	373	90	373
to P (2 − x, 1 − y, 1 − z)					to R (1 − x, y, 0.5 − z)				
N2···H21	2.5183	2.5309	−7.3	−7.4	H8···H14	2.5593	3.1797	−1.6	–
	[2.75]				H8···H15	2.3827	2.8644	−3.3	>0
O4···H20	2.7494	3.0739	−3.4	0.0		[2.40]			
	[2.72]				H15···O2	2.8829	3.1811	−1.6	>0
to Q (x, 1 − y, 0.5 + z)						[2.72]			
H3···centroid (C19–C24)	2.5341	2.7382			to S (1 − x, 1 − y, 1 − z)				
C3···centroid (C19–C24)	3.46 (3)	3.64 (3)			C3···I3	3.94 (2)	3.97 (2)	−0.7	–
H3···C24	2.6826	2.8188	−6.4	−4.7	C4···I3	4.03 (2)	3.89 (3)	–	−0.9
C17···H9	2.7720	2.9902	−3.8	−1.5		[3.68]			
	[2.90]				I3···I1	4.394 (10)	4.233 (8)	–	−0.5
N4···H9	2.7130	2.5124	−4.0	−8.4		[3.96]			
	[2.75]				to T (1.5 − x, 1.5 − y, 1 − z)				
O1···H8	2.9189	2.7812	−1.2	−2.3	I3···I4	3.856 (8)	3.968 (8)	−4.3	−2.8
	[2.72]					[3.96]			
H23···I2	3.2611	3.4235	−3.1	−1.0	to U (1.5 − x, 0.5 − y, −z)				
	[3.18]				I2···I2	3.804 (10)	3.968 (10)	−5.6	−3.0
I1···C22	3.79 (3)	3.99 (7)	−2.5	−0.9		[3.96]			
	[3.68]				to V (−0.5 + x, 0.5 + y, z)				
I1···I4	4.247 (12)	4.070 (11)	–	−2.8	I3···I2	4.897 (9)	5.104 (12)	>0	>0
	[3.96]					[3.96]			

**Table 5:** Intermolecular interaction energy between the reference and labeled [Fe(**L^Br^**)_2_]^−^ anion molecules for the major and minor ligand orientation of **1Br** at 90 K.

Bond Path	*N*_equiv_ ^a^	*N*_pair_ ^b^	Interaction Energy (kJ mol^−1^)		Bond Path	*N*_equiv_ ^a^	*N*_pair_ ^b^	Interaction Energy (kJ mol^−1^)	
Ligand Orientation			Major	Minor				Major	Minor
to P (1 − x, 1 − y, 1 − z)	2	1			to S (0.5 + x, 0.5 − y, 0.5 + z)	1	2		
Br2···Br4			−6.3	–	O1 (O2′)···H21			−7.6	−2.9
Br1′···Br4			–	−4.0	C2 (C8′)···H20			−0.1	−0.8
C3′′···Br4			–	−3.1	C14···C21			−1.6	−1.6
to Q (2 − x, 1 − y, 2 − z)	2	1			H8′···H21			–	−2.2
H14···C1′			–	−0.6	C17···Br4			−2.9	−3.0
H15···C4′			–	−6.4	Br3···Br4			−2.8	−2.8
H15···C10-C11			−6.1	–	to T (−0.5 + x, 0.5 − y, 0.5 + z)	1	2		
C7···H14			−0.0	–	Br1 (Br2′)···H23			−4.2	−2.5
Br2 (Br1′)···Br3			−0.7	−1.3	Br1···H17			−1.2	–
to R (1.5 − x, −0.5 + y, 1.5 − z)	1	2			to U (1 + x, y, z)	1	2		
N4···H9 (H3′)			−5.1	−3.4	Br3···H11 (H5′)			−6.4	−7.6
O1 (O2′)···H8 (H2′)			−6.8	−7.8	to V (1 − x, 1 − y, 2 − z)	2	1		
H3···Br4			−4.3	–	Br2 (Br1′)···H5 (H11′)			−6.1	−4.0
Br2′···Br4			–	−3.4	Br1···Br2			−2.7	–
H3 (H9′)···C22			−4.1	−7.0					

^a^ Number of equivalent intermolecular interactions within the same molecular pair. ^b^ Number of different molecular pairs with an equivalent intermolecular interaction.

## Data Availability

The crystallographic data can be obtained free of charge via https://www.ccdc.cam.ac.uk/structures/? (accessed on 16 November 2024) (or from the CCDC, 12 Union Road, Cambridge CB2 1EZ, UK; Fax: +44 1223 336033; E-mail: deposit@ccdc.cam.ac.uk).

## Supplementary Materials

The following are available online at https://www.mdpi.com/article/10.3390/molecules29225473/s1. Figure S1: Time evolution of the *χ*_M_*T* products for **1Cl** at selected temperatures; Figure S2: ^1^H NMR spectrum of **4F** in CDCl_3_.; Figure S3: ^1^H NMR spectrum of **H_2_L^F^** in CDCl_3_; Figure S4: ^1^H NMR spectrum of **5Br** in CDCl_3_; Figure S5: ^1^H NMR spectrum of **H_2_L^Br^** in CDCl_3_; Figure S6: ^1^H NMR spectrum of **5I** in CDCl_3_; Figure S7: ^1^H NMR spectrum of **H_2_L^I^** in CDCl_3_; Table S1: Crystallographic data for **1F**; Table S2: Crystallographic data for **1Cl**; Table S3: Crystallographic data for **1Br**; Table S4: Crystallographic data for **1I**; Table S5: Coordination bond length (Å) and distortion parameters Σ and Θ (°) of **1F**, **1Cl**, **1Br**, and **1I**; Table S6: Intermolecular distance (Å) for **1F**, **1Cl**, **1Br**, and **1I**; Table S7: Properties (a.u.) of the bond critical points (BCPs) along the bond paths for electron density distribution of molecular pairs of the [Fe(**L^X^**)_2_]^−^ anion with L1 and L2 and interaction energies (kJ/mol) calculated from the density of all electrons at the BCPs in **1F**, **1Cl**, **1Br**, and **1I** at 90 and 373 K; Table S8: Properties (a.u.) of the bond critical points (BCPs) along the bond paths for electron density distribution of molecular pairs of the [Fe(**L^Br^**)_2_]^−^ anion with L1′ and L2 and interaction energies (kJ/mol) calculated from the density of all electrons at the BCPs in **1Br** at 90 K.
