# Supplementary material for: The Relationship Between Spin Crossover (SCO) Behaviors, Cation and Ligand Motions, and Intermolecular Interactions in a Series of Anionic SCO Fe(III) Complexes with Halogen-Substituted Azobisphenolate Ligands
Source: Molecules. 2024 Nov 20;29(22):5473. doi: 10.3390/molecules29225473 (PMC11597063; doi:10.3390/molecules29225473)
Supplement: Supplementary file 1 [file molecules-29-05473-s001.zip › molecules-3326273-supplementary.pdf]

Supplementary Materials

The Relationship between Spin Crossover (SCO) Behaviors, Cation and Ligand Motions, and Intermolecular Interactions in a Series of Anionic SCO Fe(III) Complexes with Halogen-Substituted azobisphenolate Ligands

Mai Hirota [1], Suguru Murata [1], Takahiro Sakurai [2], Hitoshi Ohta [3], Kazuyuki Takahashi [1,*]

[1] Department of Chemistry, Graduate School of Science, Kobe University, 1-1 Rokkodai, Nada-ku, Kobe 657-8501, Japan

[2] Research Facility Center for Science and Technology, Kobe University, 1-1 Rokkodai, Nada-ku, Kobe 657-8501, Japan

[3] Molecular Photoscience Research Center, Kobe University, 1-1 Rokkodai, Nada-ku, Kobe 657-8501, Japan

Contents

Figure S1. Time evolution of the $\chi_M T$ products for **1Cl** at selected temperatures........................S3
Figure S2. ^{1}H NMR spectrum of **4F** in $CDCl_3$..S4
Figure S3. ^{1}H NMR spectrum of **H_2L^F** in $CDCl_3$...S5
Figure S4. ^{1}H NMR spectrum of **5Br** in $CDCl_3$...S6
Figure S5. ^{1}H NMR spectrum of **H_2L^{Br}** in $CDCl_3$. S7
Figure S6. ^{1}H NMR spectrum of **5I** in $CDCl_3$. S8
Figure S7. ^{1}H NMR spectrum of **H_2L^I** in $CDCl_3$...S9
Table S1. Crystallographic data for **1F**. S10
Table S2. Crystallographic data for **1Cl**. S11
Table S3. Crystallographic data for **1Br**. S13
Table S4. Crystallographic data for **1I**..S14
Table S5. Coordination bond length (Å) and distortion parameters Σ and Θ (°) of **1F**, **1Cl**, **1Br**, and **1I**. S15
Table S6. Intermolecular distance (Å) for **1F**, **1Cl**, **1Br**, and **1I**. S16
Table S7. Properties (a.u.) of the bond critical points (BCPs) along the bond paths for electron density distribution of molecular pairs of the $[Fe(\mathbf{L^X})_2]^-$ anion with L1 and L2 and interaction energies (kJ/mol) calculated from the density of all electrons at the BCPs in **1F**, **1Cl**, **1Br**, and **1I** at 90 and 373 K. S20
Table S8. Properties (a.u.) of the bond critical points (BCPs) along the bond paths for electron density distribution of molecular pairs of the $[Fe(\mathbf{L^{Br}})_2]^-$ anion with L1' and L2 and interaction energies (kJ/mol) calculated from the density of all electrons at the BCPs in **1Br** at 90 K. S28

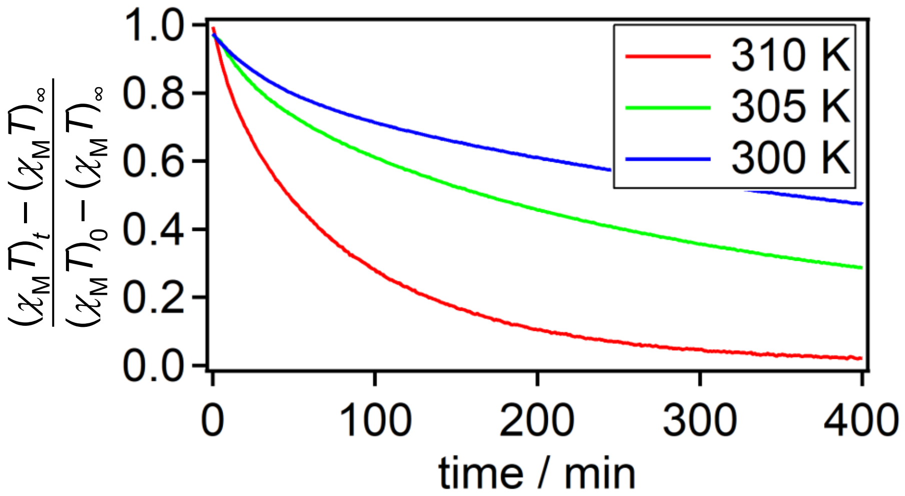

Figure S1. Time evolution of the $\chi_M T$ products for **1Cl** at selected temperatures.

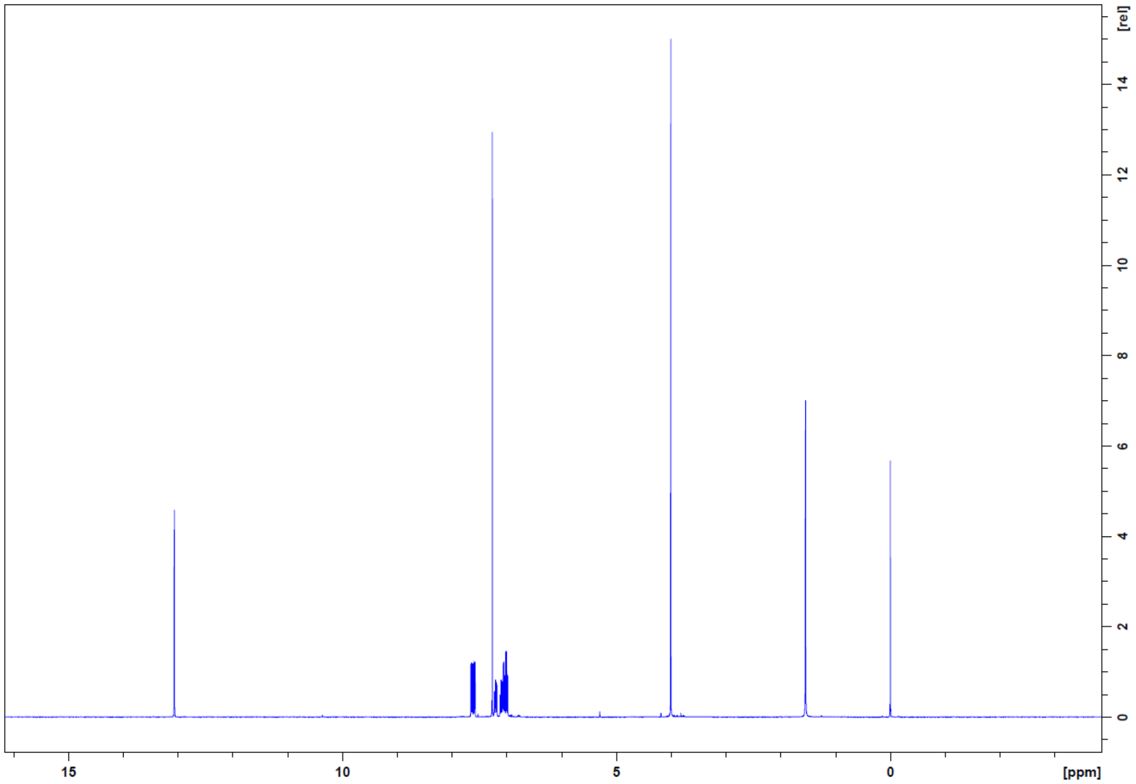

Figure S2. ^{1}H NMR spectrum of **4F** in $CDCl_3$.

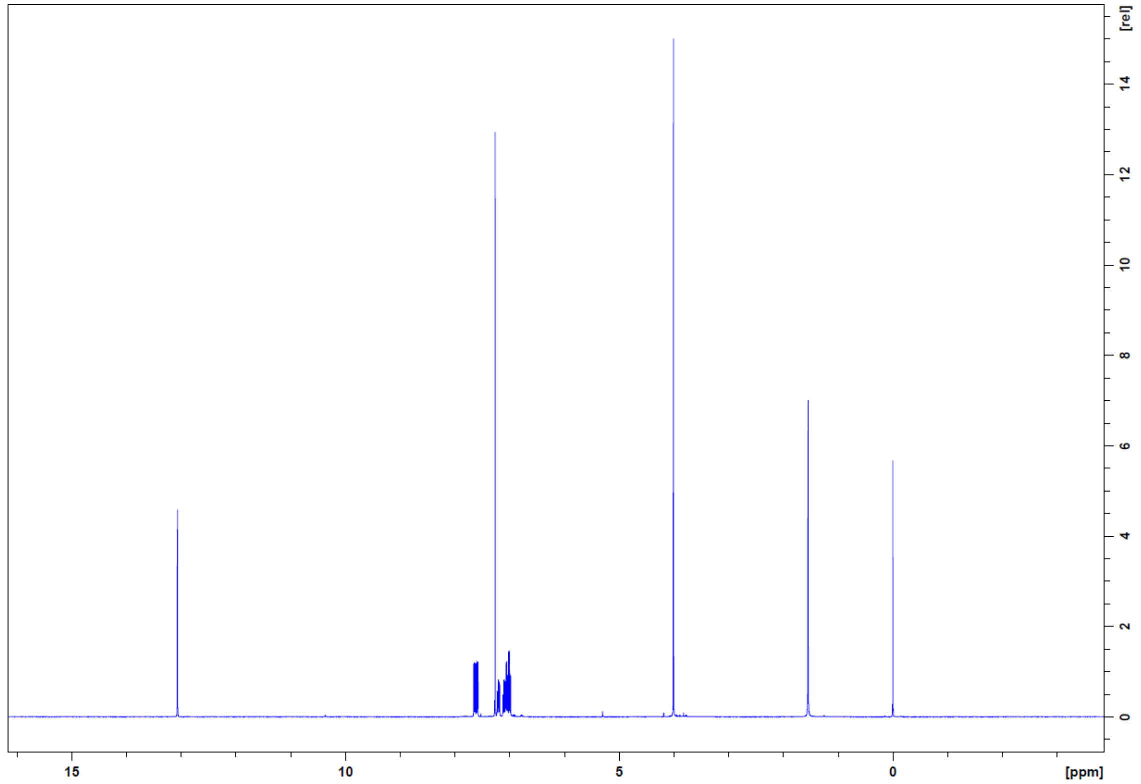

Figure S3. ^{1}H NMR spectrum of $\mathbf{H_2L^F}$ in $CDCl_3$.

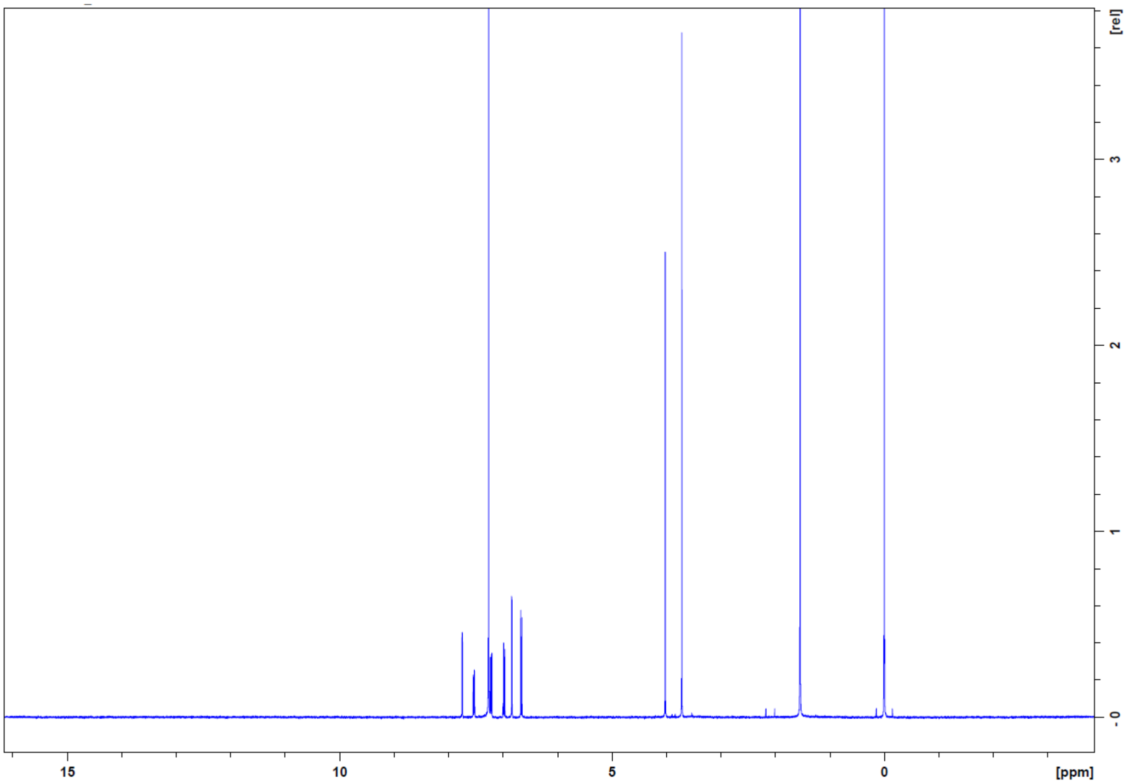

Figure S4. ^{1}H NMR spectrum of **5Br** in $CDCl_3$.

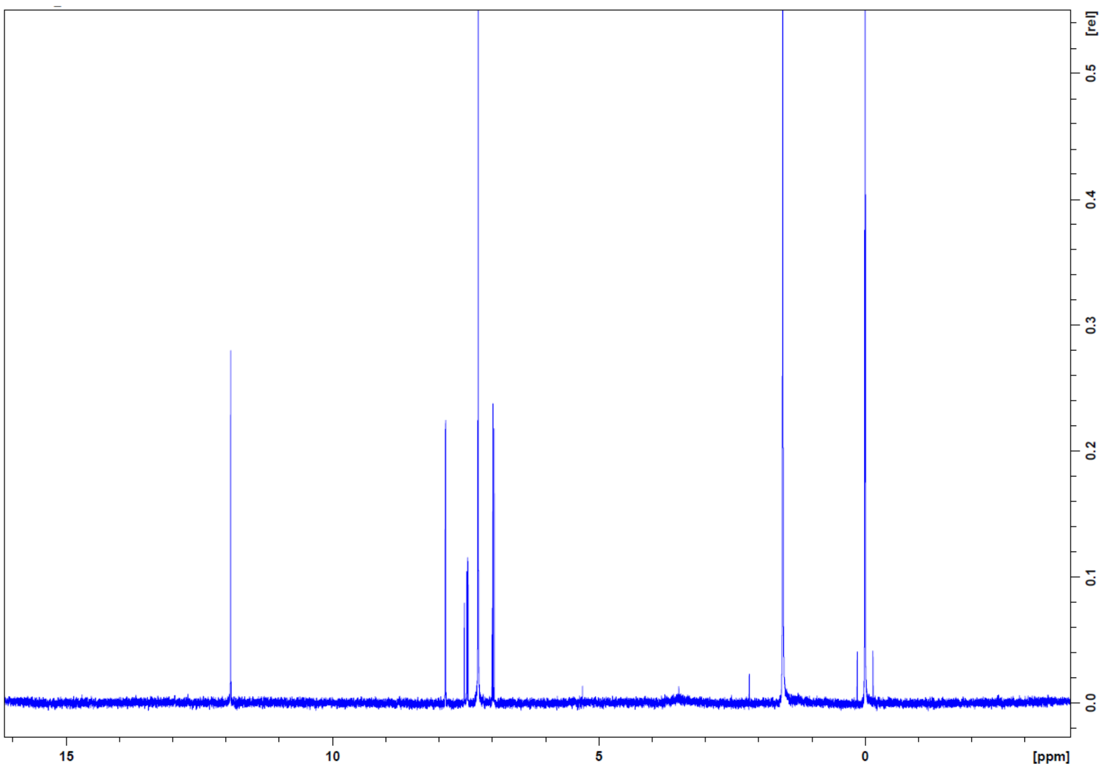

Figure S5. ^{1}H NMR spectrum of **H_2L^{Br}** in $CDCl_3$.

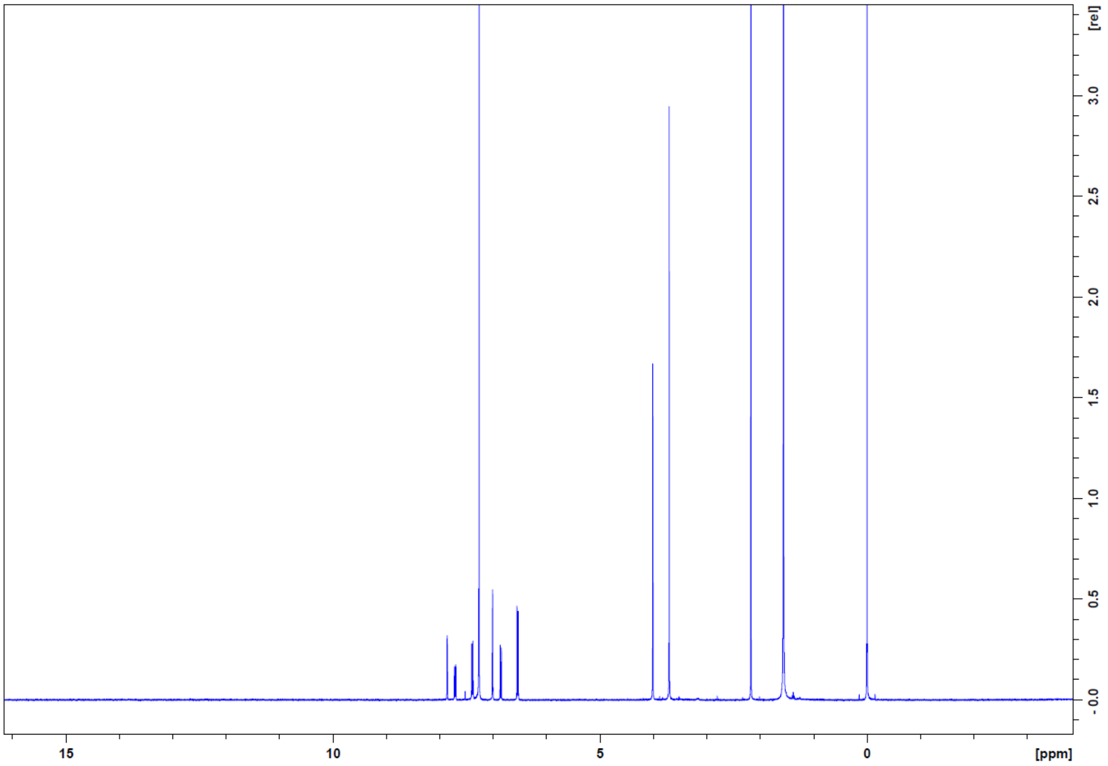

Figure S6. ^{1}H NMR spectrum of **5l** in $CDCl_3$.

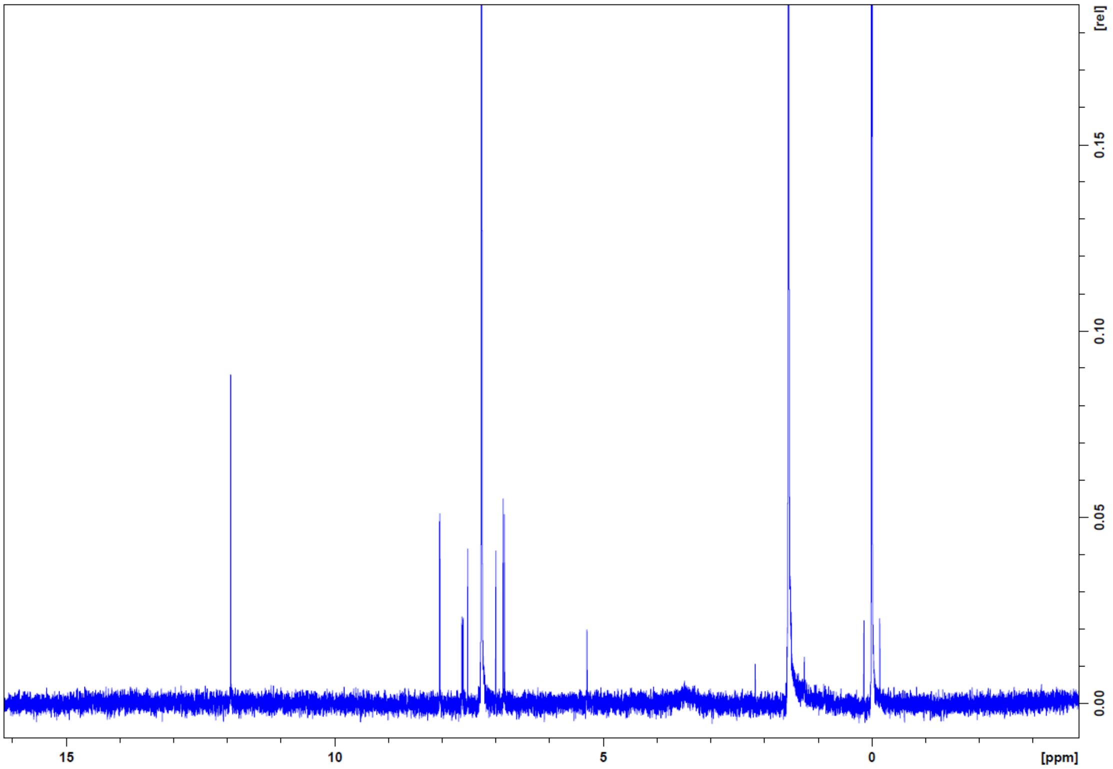

Figure S7. ^{1}H NMR spectrum of $\mathbf{H_2L^I}$ in $CDCl_3$.

Table S1. Crystallographic data for **1F**.

Formula	$C_{28}H_{24}F_4FeN_5O_4$				
Formula Weight	626.37				
Crystal No.	1				2
Color	black platelet				black platelet
Dimension / mm	0.10 × 0.10 × 0.01				0.03 × 0.01 × 0.01
T / K	90	213	293	373	90
Crystal System	monoclinic	monoclinic	monoclinic	monoclinic	monoclinic
Space Group	$P2_1/n$	$P2_1/n$	$P2_1/n$	$P2_1/n$	$P2_1/n$
a /Å	12.486(2)	12.6740(14)	12.786(2)	12.8307(13)	12.482(9)
b /Å	15.186(3)	15.1936(17)	15.201(3)	15.3187(15)	15.210(11)
c /Å	15.058(3)	15.1701(17)	15.285(3)	15.3980(15)	15.061(10)
α /°	90	90	90	90	90
β /°	107.417(3)	108.044(2)	108.625(3)	108.7080(10)	107.513(11)
γ /°	90	90	90	90	90
V /Å^3	2724.3(8)	2777.5(5)	2815.2(9)	2866.6(5)	2727.(3)
Z	4	4	4	4	4
ρ_{calcd} / g cm^{-3}	1.527	1.498	1.478	1.451	1.526
μ (Mo-Kα)	0.626	0.614	0.605	0.595	0.625
2θ_{max} /°	54.20	54.20	54.20	54.20	53.44
No. Reflections (R_{int})	15334 (0.0548)	15687 (0.0435)	15045 (0.0661)	15742 (0.0266)	14897 (0.0247)
No. Observations (I > 2.00σ(I))	6006 (4067)	6135 (3948)	6070 (3750)	6299 (3791)	5743 (4803)
No. Variables	740	740	740	740	740
$R1$ (I > 2.00σ(I))	0.0574	0.0529	0.0789	0.0518	0.0417
R (all data)	0.0951	0.0958	0.1291	0.0969	0.0520
w$R2$ (all data)	0.1347	0.1161	0.2161	0.1247	0.1051
Residual Electron Density / e Å^{-3}	0.657 −0.733	0.360 −0.389	0.812 −0.913	0.450 −0.426	0.608 −0.634
Goodness of Fit	1.021	1.048	1.060	1.019	1.047
CCDC deposit. No.	2391888	2391889	2391890	2391891	2391892

Table S2. Crystallographic data for **1Cl**.

Formula			$C_{28}H_{24}Cl_4FeN_5O_4$		
Formula Weight			692.17		
Crystal No.			1		
Color			black platelet		
Dimension / mm			0.40 × 0.10 × 0.10		
Measurement Sequence No.	1	2	3	4	5
T / K	90	293	373	90	90
Crystal System	monoclinic	monoclinic	monoclinic	monoclinic	monoclinic
Space Group	$P2_1/n$	$P2_1/n$	$P2_1/n$	$P2_1/n$	$P2_1/n$
a /Å	12.9296(13)	13.223(3)	13.4162(10)	12.9525(7)	12.9129(8)
b /Å	15.3932(16)	15.560(4)	15.6365(11)	15.4341(8)	15.3692(9)
c /Å	16.1502(16)	16.104(4)	16.2583(12)	16.0788(9)	16.2314(10)
α /°	90	90	90	90	90
β /°	111.6090(10)	111.627(3)	112.5580(10)	111.5650(10)	111.7240(10)
γ /°	90	90	90	90	90
V /Å^3	2988.4(5)	3080.1(14)	3149.8(4)	2989.3(3)	2992.5(3)
Z	4	4	4	4	4
ρ_{calcd} / g cm^{-3}	1.538	1.493	1.460	1.538	1.536
μ (Mo-Kα)	0.906	0.879	0.859	0.905	0.905
$2\theta_{max}$ /°	54.96	54.96	54.24	54.96	54.22
No. Reflections (R_{int})	17255 (0.016)	17744 (0.0226)	17757 (0.0238)	17092 (0.0130)	16669 (0.0267)
No. Observations ($I > 2.00\sigma(I)$)	6825 (6234)	7037 (5455)	6962 (4987)	6847 (6270)	6601 (6115)
No. Variables	546	546	740	710	546
$R1$ ($I > 2.00\sigma(I)$)	0.0426	0.0472	0.0410	0.0404	0.0381
R (all data)	0.0465	0.0612	0.0609	0.0440	0.0414
w$R2$ (all data)	0.1033	0.1409	0.1242	0.0983	0.0950
Residual Electron Density / e Å^{-3}	0.885 −0.674	0.425 −0.396	0.285 −0.230	0.932 −1.087	1.026 −1.320
Goodness of Fit	1.024	1.044	1.014	1.020	1.024
CCDC deposit. No.	2391893	2391894	2391895	2391896	2391897

Table S2. *Cont.*

Formula	$C_{28}H_{24}Cl_4FeN_5O_4$	
Formula Weight	692.17	
Crystal No.	1	2
Color	black platelet	
Dimension / mm	0.40 × 0.10 × 0.10	0.20 × 0.10 × 0.05
Measurement Sequence No.	6	-
T / K	90	90
Crystal System	monoclinic	monoclinic
Space Group	$P2_1/n$	$P2_1/n$
a /Å	12.9165(10)	12.891(2)
b /Å	15.3588(12)	15.352(3)
c /Å	16.2522(12)	16.247(3)
α /°	90	90
β /°	111.7350(10)	111.715(2)
γ /°	90	90
V /Å^3	2994.9(4)	2987.2(9)
Z	4	4
ρ_{calcd} / g cm^{-3}	1.535	1.539
μ (Mo-Kα)	0.904	0.906
$2\theta_{max}$ /°	54.96	54.20
No. Reflections (R_{int})	17045 (0.0160)	16640 (0.0179)
No. Observations ($I > 2.00\sigma(I)$)	6829 (6352)	6570 (6238)
No. Variables	546	546
$R1$ ($I > 2.00\sigma(I)$)	0.0376	0.0342
R (all data)	0.0404	0.0358
w$R2$ (all data)	0.0990	0.0867
Residual Electron Density / e Å^{-3}	0.980 −1.318	0.836 −1.258
Goodness of Fit	1.022	1.028
CCDC deposit. No.	2391898	2391887

Table S3. Crystallographic data for **1Br**.

Formula	$C_{28}H_{24}Br_4FeN_5O_4$				
Formula Weight	870.01				
Crystal No.	1			2	
Color	black block			black platelet	
Dimension / mm	0.15 × 0.15 × 0.02			0.20 × 0.20 × 0.02	
Measurement Sequence No.	1	2	3	1	2
T / K	90	373	90	90	293
Crystal System	monoclinic	monoclinic	monoclinic	monoclinic	monoclinic
Space Group	$P2_1/n$	$P2_1/n$	$P2_1/n$	$P2_1/n$	$P2_1/n$
a /Å	13.1884(12)	13.588(2)	13.1861(12)	13.176(2)	13.52(2)
b /Å	15.4629(14)	15.748(3)	15.4499(14)	15.450(3)	15.75(3)
c /Å	16.5077(15)	16.567(3)	16.4960(15)	16.514(3)	16.42(3)
α /°	90	90	90	90	90
β /°	112.3200(10)	113.142(3)	112.3560(10)	112.378(2)	111.773(19)
γ /°	90	90	90	90	90
V /Å^3	3114.2(5)	3259.8(10)	3108.0(5)	3108.6(9)	3247.(9)
Z	4	4	4	4	4
ρ_{calcd} / g cm^{-3}	1.856	1.773	1.859	1.859	1.779
μ (Mo-Kα)	5.659	5.406	5.670	5.669	5.426
$2\theta_{max}$ /°	54.20	50.04	54.96	54.20	54.20
No. Reflections (R_{int})	17339 (0.0135)	14790 (0.0630)	17822 (0.019)	17429 (0.0249)	18123 (0.0420)
No. Observations ($I > 2.00\sigma(I)$)	6831 (6166)	5695 (2920)	7132 (6153)	6824 (5770)	7127 (4506)
No. Variables	546	750	546	546	546
$R1$ ($I > 2.00\sigma(I)$)	0.0235	0.0567	0.0264	0.0273	0.0460
R (all data)	0.0280	0.1181	0.0343	0.0358	0.0863
w$R2$ (all data)	0.0551	0.1640	0.0603	0.0654	0.1227
Residual Electron Density / e Å^{-3}	1.048 −0.841	0.439 −0.429	1.211 −0.922	0.946 −0.820	1.039 −0.772
Goodness of Fit	1.028	1.027	1.029	1.027	1.029
CCDC deposit. No.	2391899	2391900	2391901	2391902	2391903

Table S4. Crystallographic data for **1I**.

Formula		$C_{28}H_{24}FeI_4N_5O_4$	
Formula Weight		1057.97	
Crystal No.	1	2	3
Color	black needle	black block	black block
Dimension / mm	0.20×0.03×0.02	0.20×0.10×0.05	0.10×0.06×0.04
T / K	90	295	373
Crystal System	monoclinic	monoclinic	monoclinic
Space Group	$C2/c$	$C2/c$	$C2/c$
a /Å	17.370(6)	17.589(4)	17.674(3)
b /Å	26.685(9)	27.169(6)	27.330(5)
c /Å	16.273(5)	16.404(4)	16.454(3)
α /°	90	90	90
β /°	116.321(3)	117.137(4)	117.146(2)
γ /°	90	90	90
V /Å^3	6761.(4)	6976.(3)	7072.(2)
Z	8	8	8
ρ_{calcd} / g cm^{-3}	2.079	2.015	1.987
μ (Mo-Kα)	4.138	4.011	3.956
$2\theta_{max}$ /°	54.96	50.06	53.46
No. Reflections (R_{int})	18925 (0.0430)	15664 (0.0395)	19561 (0.0214)
No. Observations ($I > 2.00\sigma(I)$)	7717 (3482)	6062 (3591)	7518 (3346)
No. Variables	754	705	705
$R1$ ($I > 2.00\sigma(I)$)	0.0635	0.0689	0.0455
R (all data)	0.1483	0.1071	0.1106
w$R2$ (all data)	0.2133	0.2285	0.1545
Residual Electron Density / e Å^{-3}	1.498 −0.979	1.710 −0.793	0.500 −0.371
Goodness of Fit	1.042	1.057	0.995
CCDC deposit. No.	2391904	2391905	2391906

Table S5. Coordination bond length (Å) and distortion parameters Σ and Θ (°) of **1F**, **1Cl**, **1Br**, and **1I**.

**Complex**			**1F**[a]		
Crystal No.			1		2
Temp. / K	90	213	293	373	90
Fe1-O1	1.909(4)	1.926(5)	1.961(8)	1.961(7)	1.909(3)
Fe1-O2	1.908(4)	1.925(5)	1.932(9)	1.954(7)	1.909(3)
Fe1-N1	1.945(4)	2.050(3)	2.145(5)	2.164(3)	1.954(3)
Fe1-O3	1.912(3)	1.932(4)	1.959(6)	1.968(5)	1.908(2)
Fe1-O4	1.913(4)	1.933(4)	1.941(6)	1.951(5)	1.914(3)
Fe1-N3	1.927(3)	2.026(4)	2.123(5)	2.143(3)	1.927(3)
Σ	42.4(8)	66.7(12)	89.8(13)	98.5(10)	40.1(6)
Θ	48.8(9)	80.7(13)	135.7(14)	157.5(11)	47.5(7)

**Complex**				**1Cl**				
Crystal No.				1				2
Seq.				I	II	III	IV	
Temp. / K	90	293	373	90	90	90	90	90
Fe1-O1	1.941(2)	1.930(3)	1.981(8)	1.934(2)	1.9376(17)	1.9340(16)	1.9385(17)	1.9366(15)
Fe1-O2	1.857(2)	1.886(3)	1.920(9)	1.865(2)	1.8602(18)	1.8624(17)	1.8600(17)	1.8609(16)
Fe1-N1	1.9189(19)	1.960(2)	2.128(3)	1.921(2)	1.9131(16)	1.9124(16)	1.9135(16)	1.9078(15)
Fe1-O3	1.9267(14)	1.9273(18)	1.967(3)	1.9248(15)	1.9257(13)	1.9255(13)	1.9263(12)	1.9246(12)
Fe1-O4	1.8735(15)	1.8878(18)	1.940(3)	1.8740(15)	1.8752(13)	1.8752(13)	1.8752(13)	1.8749(12)
Fe1-N3	1.9103(14)	1.9512(18)	2.107(2)	1.9126(14)	1.9073(14)	1.9076(14)	1.9080(13)	1.9039(13)
Σ	43.1(5)	51.5(9)	76.4(11)	50.8(7)	33.5(4)	32.1(4)	33.5(3)	31.2(3)
Θ	54.5(5)	64.5(10)	125.7(12)	63.8(7)	42.1(4)	42.0(4)	41.9(4)	39.4(3)

**Complex**			**1Br**				**1I**	
Crystal No.		1		2		1	2	3
			V					
Temp. / K	90	373	90	90	293	90	295	373
Fe1-O1	1.935(2)	1.961(5)	1.933(2)	1.931(2)	1.947(5)	2.021(9)	2.062(11)	2.010(8)
Fe1-O2	1.857(2)	1.924(6)	1.854(2)	1.855(2)	1.867(5)	1.846(10)	1.866(11)	1.919(9)
Fe1-N1	1.9050(19)	2.075(5)	1.904(2)	1.903(2)	1.915(4)	2.033(7)	2.100(7)	2.139(4)
Fe1-O3	1.9197(15)	1.960(5)	1.9183(16)	1.9194(17)	1.932(4)	2.021(9)	2.062(11)	2.010(9)
Fe1-O4	1.8833(15)	1.925(6)	1.8815(16)	1.8816(17)	1.891(3)	1.846(10)	1.866(11)	1.917(9)
Fe1-N3	1.8985(17)	2.074(4)	1.8989(18)	1.899(2)	1.915(4)	2.031(7)	2.099(7)	2.139(4)
Σ	27.1(5)	71.8(18)	27.4(5)	27.4(4)	31.9(7)	76(2)	82(3)	100.2(17)
Θ	36.9(5)	111.3(17)	37.3(5)	37.1(5)	42.3(8)	83(3)	127(3)	142.3(18)

[a] Coordination geometry with ligands L1' and L2.

Table S6. Intermolecular distance (Å) for **1F**, **1Cl**, **1Br**, and **1I**.

**Complex**	**1F**				
Crystal No.	1				2
Temp. / K	90	213	293	373	90
within a 2D network					
to P (1−x, 1−y, 1−z)					
F2⋯F4	3.032(11)	3.000(14)	3.00(2)	3.06(2)	3.014(9)
to Q (2−x, 1−y, 2−z)					
H15⋯centroid(C7-C12)	2.5945	2.6297	2.6651	2.6917	2.5989
C15⋯centroid(C7-C12)	3.520(14)	3.52(2)	3.54(3)	3.55(2)	3.523(11)
H15⋯C7	2.6602	2.7650	2.8303	2.9334	2.6752
H15⋯C8	2.7242	2.7727	2.9277	2.9433	2.7525
H15⋯C10	3.2131	3.1652	3.1498	3.1066	3.1828
O2⋯H14	2.6713	2.6174	2.6634	2.6135	2.6665
O3⋯H14	2.8543	2.8953	3.1262	3.0646	2.852
C7⋯H14	3.0840	2.9885	2.9314	2.8946	3.1072
C8⋯H14	3.3794	3.3254	3.2590	3.2741	3.4032
F2⋯F3	4.851(11)	4.903(15)	4.94(3)	4.96(2)	4.854(8)
to R (1.5−x, −0.5+y, 1.5−z)					
N4⋯H9	2.6694	2.6567	2.6793	2.5993	2.6617
O1⋯H8	2.7085	2.5638	2.6753	2.6326	2.6997
H3⋯F4	3.2084	3.3502	3.4371	3.4518	3.2000
H3⋯C22	2.9022	2.8563	2.8393	2.8647	2.8668
to S (0.5+x, 0.5−y, 0.5+z)					
O1⋯H21	2.5936	2.7834	2.9023	2.9647	2.613
C2⋯H20	2.7542	2.9562	3.0017	3.0593	2.7686
C14⋯C21	3.585(16)	3.68(2)	3.71(3)	3.86(3)	3.595(11)
C15⋯C22	3.500(13)	3.52(2)	3.63(3)	3.66(2)	3.512(10)
C16⋯C22	3.300(10)	3.360(15)	3.44(2)	3.48(2)	3.307(8)
C16⋯F4	3.210(8)	3.268(12)	3.309(17)	3.336(17)	3.204(6)
C17⋯F4	3.245(7)	3.338(10)	3.399(15)	3.463(13)	3.243(5)
F3⋯F4	3.250(7)	3.290(10)	3.304(16)	3.343(15)	3.244(5)
between 2D networks					
to T (−0.5+x, 0.5−y, 0.5+z)					
F1⋯H23	2.8439	2.9723	3.0034	2.9519	2.8495
F1⋯H17	2.6203	2.6581	2.7733	2.7648	2.6158
to U (1+x, y, z)					
F3⋯H11	2.4638	2.4863	2.4989	2.5917	2.4176
to V (1−x, 1−y, 2−z)					
F2⋯H5	2.6888	2.7478	2.8332	2.7849	2.6665
F1…F2	4.224(15)	4.33(2)	4.45(4)	4.45(3)	4.230(11)

Table S6. *Cont.*

Complex	1Cl							
Crystal No.				1				2
				I	II	III	IV	
Temp. / K	90	293	373	90	90	90	90	90
within a 2D network								
to P (1−x, 1−y, 1−z)								
Cl2⋯Cl4	3.3455(14)	3.387(3)	3.373(7)	3.3170(19)	3.3688(10)	3.3763(9)	3.3692(10)	3.3732(10)
to Q (2−x, 1−y, 2−z)								
H15⋯centroid(C7-C12)	2.4614	2.5615	2.6825	2.4671	2.4537	2.4517	2.4523	2.4482
C15⋯centroid(C7-C12)	3.360(3)	3.426(8)	3.492(15)	3.366(5)	3.351(3)	3.350(3)	3.350(3)	3.346(2)
H15⋯C7	2.8543	3.0010	3.1998	2.8647	2.8556	2.8542	2.8541	2.8537
H15⋯C8	2.9408	3.0300	3.0986	2.9499	2.9161	2.9058	2.9119	2.9054
H15⋯C10	2.7526	2.7657	2.7908	2.7627	2.7600	2.7639	2.7609	2.7587
O2⋯H14	2.9450	2.9648	3.1216	2.9259	2.9373	2.9417	2.9372	2.9385
O3⋯H14	3.0544	3.2501	3.6018	3.0101	3.1277	3.1406	3.1302	3.1418
C7⋯H14	3.0785	3.0854	3.1296	3.0808	3.0577	3.0549	3.0596	3.0538
C8⋯H14	3.3040	3.3654	3.2975	3.3074	3.2923	3.2925	3.2948	3.2822
Cl2⋯Cl3	4.1571(15)	4.234(3)	4.320(8)	4.156(2)	4.1771(11)	4.1812(9)	4.1776(10)	4.1824(10)
to R (1.5−x, −0.5+y, 1.5−z)								
N4⋯H9	2.6458	2.6433	2.5469	2.6365	2.6467	2.6503	2.6516	2.6515
O1⋯H8	2.4720	2.5252	2.5645	2.5164	2.4683	2.4691	2.4715	2.4686
H3⋯Cl4	3.0082	3.1582	3.5009	3.0683	2.9441	2.9378	2.9473	2.921
H3⋯C22	2.8315	2.9172	2.9380	2.8466	2.8315	2.8257	2.8348	2.8235
to S (0.5+x, 0.5−y, 0.5+z)								
O1⋯H21	2.5278	2.6448	2.9213	2.5632	2.4873	2.4848	2.4845	2.4723
C2⋯H20	2.9676	3.1013	2.9895	2.9714	2.9835	2.9847	2.9862	2.9929
C14⋯C21	3.568(3)	3.628(5)	3.628(16)	3.603(6)	3.533(3)	3.523(3)	3.529(3)	3.516(3)
C15⋯C22	3.615(3)	3.665(4)	3.655(12)	3.627(8)	3.611(3)	3.610(3)	3.611(3)	3.608(3)
C16⋯C22	3.452(3)	3.551(4)	3.672(16)	3.446(7)	3.464(3)	3.463(3)	3.465(3)	3.465(3)
C16⋯Cl4	3.542(2)	3.598(3)	3.701(11)	3.538(5)	3.554(2)	3.5523(19)	3.5539(19)	3.5536(19)
C17⋯Cl4	3.491(5)	3.560(3)	3.715(8)	3.483(3)	3.5090(19)	3.5085(18)	3.5085(18)	3.5086(17)
Cl3⋯Cl4	3.6498(9)	3.7626(17)	3.813(6)	3.6439(14)	3.6739(8)	3.6761(8)	3.6752(8)	3.6744(8)
between 2D networks								
to T (−0.5+x, 0.5−y, 0.5+z)								
Cl1⋯H23	2.9087	3.1098	3.0831	2.9298	2.8976	2.8963	2.8995	2.8875
Cl1⋯H17	3.0525	3.0417	3.1530	3.0244	3.0964	3.1121	3.1007	3.1116
to U (1+x, y, z)								
Cl3⋯H11	2.8406	2.9664	2.9187	2.8832	2.8313	2.8274	2.8344	2.8274
to V (1−x, 1−y, 2−z)								
Cl2⋯H5	2.7572	2.8121	2.8543	2.7198	2.8064	2.8132	2.8035	2.8140
Cl1⋯Cl2	3.770(3)	4.004(6)	4.292(11)	3.787(4)	3.777(2)	3.796(2)	3.780(2)	3.7736(18)

Table S6. *Cont.*

**Complex**			**1Br**		
Crystal No.		1		2	
			V		
Temp. / K	90	373	90	90	293
within a 2D network					
to P (1−x, 1−y, 1−z)					
Br2···Br4	3.5088(5)	3.534(7)	3.5062(6)	3.5083(8)	3.577(6)
to Q (2−x, 1−y, 2−z)					
H15···centroid(C7-C12)	2.4615	2.6770	2.4556	2.4524	2.5823
C15···centroid(C7-C12)	3.357(4)	3.48(2)	3.350(4)	3.349(5)	3.436(8)
H15···C7	2.9107	3.2866	2.9013	2.9000	3.0926
H15···C8	2.9368	3.3233	2.9367	2.9320	3.0786
H15···C10	2.7327	2.6620	2.7212	2.7226	2.7595
O2···H14	3.0635	3.1427	3.0560	3.0598	3.1831
O3···H14	3.2414	3.7105	3.2367	3.2569	3.5034
C7···H14	3.0963	3.1005	3.0857	3.0955	3.1426
C8···H14	3.2869	3.1662	3.2831	3.2742	3.2707
Br2···Br3	4.1230(5)	4.304(8)	4.1195(6)	4.1208(7)	4.234(5)
to R (1.5−x, −0.5+y, 1.5−z)					
N4···H9	2.6371	2.6960	2.6457	2.6389	2.7186
O1···H8	2.4892	2.5238	2.4898	2.4975	2.5891
H3···Br4	3.0382	3.4622	3.0551	3.0349	3.1819
H3···C22	2.8377	2.8304	2.7834	2.8337	2.8894
to S (0.5+x, 0.5−y, 0.5+z)					
O1···H21	2.4916	2.8781	2.4873	2.4861	2.5976
C2···H20	3.1384	3.2637	3.1027	3.1389	3.2795
C14···C21	3.514(3)	3.79(2)	3.513(3)	3.497(4)	3.584(9)
C15···C22	3.653(3)	3.81(3)	3.650(3)	3.652(4)	3.726(9)
C16···C22	3.575(3)	3.83(3)	3.571(3)	3.572(4)	3.714(8)
C16···Br4	3.668(2)	3.83(2)	3.666(2)	3.667(3)	3.753(8)
C17···Br4	3.605(2)	3.839(11)	3.600(2)	3.606(3)	3.692(7)
Br3···Br4	3.8714(4)	4.096(6)	3.8691(5)	3.8684(6)	4.064(6)
between 2D networks					
to T (−0.5+x, 0.5−y, 0.5+z)					
Br1···H23	3.0390	3.4138	3.0415	3.0384	3.2926
Br1···H17	3.2614	3.0868	3.2616	3.2658	3.1514
to U (1+x, y, z)					
Br3···H11	2.8784	2.9790	2.8790	2.8763	3.0540
to V (1−x, 1−y, 2−z)					
Br2···H5	2.8570	3.0876	2.8593	2.8566	2.9411
Br1···Br2	3.8522(16)	4.190(11)	3.8535(17)	3.8524(18)	4.030(7)

Table S6. *Cont.*

**Complex**		**1I**	
Crystal No.	1	2	3
Temp. / K	90	295	373
within a 2D network			
to P (2−x, 1−y, 1−z)			
N2…H21	2.5183	2.6939	2.5309
O4…H20	2.7494	3.2367	3.0739
to Q (x, 1−y, 0.5+z)			
H3…centroid(C19-C24)	2.5341	2.5821	2.7382
C3…centroid(C19-C24)	3.46(3)	3.49(4)	3.64(3)
H3…C24	2.6826	2.7984	2.8188
C17…H9	2.7720	2.7133	2.9902
N4…H9	2.7130	2.7259	2.5124
O1…H8	2.9189	3.0258	2.7812
H23…I2	3.2611	3.4568	3.4235
I1…C22	3.79(3)	3.82(3)	3.99(7)
I1…I4	4.247(12)	4.237(12)	4.070(11)
to R (1−x, y, 0.5−z)			
H8…H14	2.5593	2.7647	3.1797
H8…H15	2.3827	2.6935	2.8644
H15…O2	2.8829	3.1369	3.1811
to S (1−x, 1−y, 1−z)			
C3…I3	3.94(2)	4.12(3)	3.97(2)
C4…I3	4.03(2)	4.19(2)	3.89(3)
I3…I1	4.394(10)	4.380(11)	4.233(8)
between 2D networks			
to T (1.5−x, 1.5−y, 1−z)			
I3…I4	3.856(8)	3.865(9)	3.968(8)
to U (1.5−x, 0.5−y, −z)			
I2…I2	3.804(10)	4.047(13)	3.968(10)
to V (−0.5+x, 0.5+y, z)			
I3…I2	4.891(9)	4.873(14)	5.104(12)

Table S7. Properties (a.u.) of the bond critical points (BCPs) along the bond paths for electron density distribution of molecular pairs of the $[Fe(\mathbf{L}^X)_2]^-$ anion with L1 and L2 and interaction energies (kJ/mol) calculated from the density of all electrons at the BCPs in **1F**, **1Cl**, **1Br**, and **1I** at 90 and 373 K.

**1F** at 90 K							
**Bond Path**	N_{equiv} [a]	$\rho(r)$ [b]	$\nabla^2\rho(r)$ [c]	H_b [d]	$V(r)$ [e]	$G(r)$ [f]	E_{int} [g]
within a 2D network							
to P (1−x, 1−y, 1−z)							
F2···F4	2	0.0039	0.0201	0.0010	−0.0031	0.0041	−0.5
C9···F4	2	0.0039	0.0172	0.0010	−0.0022	0.0033	−0.5
C9···H21	2	0.0047	0.0194	0.0013	−0.0022	0.0035	−1.3
C20···C20	1	0.0001	0.0004	0.0000	0.0000	0.0001	3.0
to Q (2−x, 1−y, 2−z)							
H15···C7	2	0.0097	0.0349	0.0016	−0.0054	0.0071	−5.9
H14···O3	2	0.0051	0.0220	0.0014	−0.0028	0.0041	−1.6
H14···O2	2	0.0071	0.0274	0.0012	−0.0044	0.0056	−3.5
to R (1.5−x, −0.5+y, 1.5−z)							
N4···H9	1	0.0089	0.0336	0.0015	−0.0054	0.0069	−5.2
O1···H8	1	0.0075	0.0268	0.0011	−0.0045	0.0056	−3.9
H2···C20	1	0.0009	0.0029	0.0002	−0.0003	0.0005	2.3
H3···C22	1	0.0059	0.0210	0.0012	−0.0029	0.0041	−2.4
to S (0.5+x, 0.5−y, 0.5+z)							
O1···H21	1	0.0088	0.0318	0.0012	−0.0055	0.0067	−5.1
C2···H20	1	0.0069	0.0267	0.0015	−0.0037	0.0052	−3.3
C14···C21	1	0.0043	0.0132	0.0009	−0.0016	0.0025	−0.9
H3···C10	1	0.0001	0.0004	0.0000	0.0000	0.0001	3.0
C16···C22	1	0.0058	0.0198	0.0012	−0.0026	0.0038	−2.3
C16···F4	1	0.0055	0.0222	0.0012	−0.0032	0.0044	−2.0
C18···C21	1	0.0039	0.0145	0.0011	−0.0015	0.0026	−0.6
H2···H21	1	0.0038	0.0192	0.0015	−0.0018	0.0033	−0.4
between 2D networks							
to T (0.5+x, 0.5−y, −0.5+z)							
H23···F1	1	0.0040	0.0174	0.0009	−0.0027	0.0035	−0.7
H17···F1	1	0.0062	0.0270	0.0012	−0.0044	0.0056	−2.7
N4···F1	1	0.0036	0.0145	0.0007	−0.0021	0.0029	−0.3
to U (1+x, y, z)							
F3···H11	1	0.0097	0.0425	0.0018	−0.0071	0.0089	−5.9
to V (1−x, 1−y, 2−z)							
H11···H11	1	0.0040	0.0186	0.0016	−0.0016	0.0031	−0.6
H5···F2	2	0.0057	0.0269	0.0014	−0.0039	0.0053	−2.2

[a] Number of equivalent bond paths (N_{equiv}). [b] Densities of all electrons ($\rho(r)$). [c] Laplacian of electron density ($\rho(r)$). [d] Energy density (H_b). [e] Potential energy density ($V(r)$). [f] Lagrangian kinetic energy ($G(r)$) (a.u.). [g] Interaction energies (E_{int}) (kJ/mol).

Table S7. *Cont.*

Bond Path	N_{equiv} [a]	$\rho(r)$ [b]	$\nabla^2\rho(r)$ [c]	H_b [d]	$V(r)$ [e]	$G(r)$ [f]	E_{int} [g]
**1F** at 373 K							
within a 2D network							
to P (1−x, 1−y, 1−z)							
F2···F4	2	0.0037	0.0188	0.0009	−0.0029	0.0038	−0.3
C20···C20	1	0.0001	0.0004	0.0000	0.0000	0.0001	3.0
H21···C9	2	0.0039	0.0140	0.0009	−0.0017	0.0026	−0.5
to Q (2−x, 1−y, 2−z)							
H15···C8	2	0.0059	0.0233	0.0014	−0.0030	0.0044	−2.4
O3···O3	1	0.0012	0.0046	0.0003	−0.0006	0.0009	2.0
H14···O2	2	0.0085	0.0310	0.0012	−0.0054	0.0066	−4.8
to R (1.5−x, −0.5+y, 1.5−z)							
N4···H9	1	0.0097	0.0354	0.0014	−0.0060	0.0074	−6.0
O1···H8	1	0.0084	0.0309	0.0012	−0.0053	0.0065	−4.8
H3···C22	1	0.0076	0.0263	0.0013	−0.0040	0.0053	−4.0
to S (0.5+x, 0.5−y, 0.5+z)							
O1···H21	1	0.0041	0.0154	0.0008	−0.0022	0.0030	−0.7
C2···H20	1	0.0038	0.0141	0.0010	−0.0016	0.0026	−0.4
H3···C10	1	0.0000	0.0002	0.0000	0.0000	0.0000	3.1
C16···C22	1	0.0041	0.0137	0.0009	−0.0017	0.0025	−0.7
C16···F4	1	0.0039	0.0157	0.0009	−0.0022	0.0031	−0.6
C18···C21	1	0.0024	0.0079	0.0006	−0.0009	0.0014	0.9
H2···H21	1	0.0024	0.0118	0.0010	−0.0010	0.0020	0.9
between 2D networks							
to T (0.5+x, 0.5−y, −0.5+z)							
H23···F1	1	0.0032	0.0143	0.0008	−0.0020	0.0028	0.1
H17···F1	1	0.0045	0.0202	0.0010	−0.0031	0.0041	−1.1
N4···F1	1	0.0027	0.0104	0.0006	−0.0015	0.0020	0.6
to U (1+x, y, z)							
F3···H11	1	0.0073	0.0319	0.0014	−0.0051	0.0066	−3.7
to V (1−x, 1−y, 2−z)							
H11···H11	1	0.0026	0.0120	0.0011	−0.0009	0.0019	0.6
H5···F2	2	0.0040	0.0187	0.0010	−0.0027	0.0037	−0.6

[a] Number of equivalent bond paths (N_{equiv}). [b] Densities of all electrons ($\rho(r)$). [c] Laplacian of electron density ($\rho(r)$). [d] Energy density (H_b). [e] Potential energy density ($V(r)$). [f] Lagrangian kinetic energy ($G(r)$) (a.u.). [g] Interaction energies (E_{int}) (kJ/mol).

Table S7. *Cont.*

Bond Path	N_{equiv} [a]	$\rho(r)$ [b]	$\nabla^2\rho(r)$ [c]	H_b [d]	$V(r)$ [e]	$G(r)$ [f]	E_{int} [g]
**1Cl** at 90 K							
within a 2D network							
to P (1−x, 1−y, 1−z)							
Cl2···Cl4	2	0.0091	0.0334	0.0016	−0.0052	0.0068	−5.4
C21···C21	1	0.0001	0.0003	0.0000	0.0000	0.0000	3.0
H21···C9	2	0.0028	0.0115	0.0009	−0.0011	0.0020	0.5
to Q (2−x, 1−y, 2−z)							
H15···C10	2	0.0092	0.0324	0.0013	−0.0054	0.0068	−5.5
H14···O3	2	0.0034	0.0142	0.0010	−0.0016	0.0026	−0.1
H14···O2	2	0.0039	0.0163	0.0009	−0.0022	0.0031	−0.6
Cl2···Cl3	2	0.0014	0.0014	0.0014	0.0014	0.0014	0.9
to R (1.5−x, −0.5+y, 1.5−z)							
N4···H9	1	0.0089	0.0329	0.0015	−0.0053	0.0068	−5.2
O1···H8	1	0.0109	0.0414	0.0016	−0.0072	0.0088	−7.1
H2···C20	1	0.0009	0.0029	0.0002	−0.0003	0.0005	2.3
H3···C22	1	0.0070	0.0246	0.0011	−0.0040	0.0051	−3.4
H17···Cl2	1	0.0029	0.0107	0.0007	−0.0013	0.0020	0.4
to S (0.5+x, 0.5−y, 0.5+z)							
O1···H21	1	0.0105	0.0362	0.0012	−0.0067	0.0079	−6.7
C2···H20	1	0.0048	0.0176	0.0011	−0.0022	0.0033	−1.4
C14···C21	1	0.0047	0.0139	0.0009	−0.0017	0.0026	−1.3
H3···C10	1	0.0001	0.0003	0.0000	0.0000	0.0001	3.0
C16···C22	1	0.0051	0.0167	0.0009	−0.0024	0.0033	−1.7
C17···Cl4	1	0.0062	0.0210	0.0012	−0.0029	0.0041	−2.7
Cl3···Cl4	1	0.0059	0.0198	0.0010	−0.0030	0.0040	−2.4
H2···H21	1	0.0033	0.0167	0.0013	−0.0015	0.0028	0.0
between 2D networks							
to T (0.5+x, 0.5−y, −0.5+z)							
H23···Cl1	1	0.0078	0.0256	0.0010	−0.0044	0.0054	−4.2
H17···Cl1	1	0.0052	0.0189	0.0010	−0.0028	0.0037	−1.8
N4···Cl1	1	0.0037	0.0119	0.0007	−0.0017	0.0023	−0.3
to U (1+x, y, z)							
Cl3···H11	1	0.0085	0.0299	0.0013	−0.0049	0.0062	−4.8
Cl3···H5	1	0.0023	0.0091	0.0006	−0.0010	0.0016	1.0
Cl3···N2	1	0.0034	0.0116	0.0007	−0.0016	0.0022	−0.0
to V (1−x, 1−y, 2−z)							
H11···H11	1	0.0015	0.0070	0.0006	−0.0005	0.0011	1.7
H5···Cl2	2	0.0098	0.0355	0.0014	−0.0060	0.0074	−6.1
Cl1···Cl2	2	0.0046	0.0144	0.0008	−0.0021	0.0029	−1.2

[a] Number of equivalent bond paths (N_{equiv}). [b] Densities of all electrons ($\rho(r)$). [c] Laplacian of electron density ($\rho(r)$). [d] Energy density (H_b). [e] Potential energy density ($V(r)$). [f] Lagrangian kinetic energy ($G(r)$) (a.u.). [g] Interaction energies (E_{int}) (kJ/mol).

Table S7. *Cont.*

Bond Path	N_{equiv} [a]	$\rho(r)$ [b]	$\nabla^2\rho(r)$ [c]	H_b [d]	$V(r)$ [e]	$G(r)$ [f]	E_{int} [g]
**1Cl** at 373 K							
within a 2D network							
to P (1−x, 1−y, 1−z)							
Cl2···Cl4	2	0.0086	0.0315	0.0015	−0.0048	0.0063	−4.9
C20···C20	1	0.0001	0.0003	0.0000	0.0000	0.0000	3.0
H21···C9	2	0.0027	0.0094	0.0007	−0.0010	0.0017	0.6
to Q (2−x, 1−y, 2−z)							
H15···C10	2	0.0087	0.0288	0.0011	−0.0049	0.0061	−5.0
H14···C7	2	0.0030	0.0136	0.0010	−0.0015	0.0024	0.3
Cl2···Cl3	2	0.0018	0.0053	0.0003	−0.0008	0.0011	1.4
to R (1.5−x, −0.5+y, 1.5−z)							
N4···H9	1	0.0100	0.0354	0.0013	−0.0062	0.0075	−6.2
O1···H8	1	0.0092	0.0335	0.0012	−0.0059	0.0071	−5.5
H2···C20	1	0.0009	0.0030	0.0002	−0.0003	0.0005	2.3
H3···C22	1	0.0066	0.0218	0.0010	−0.0034	0.0044	−3.1
H17···Cl2	1	0.0016	0.0061	0.0005	−0.0006	0.0011	1.6
Cl1···Cl4	1	0.0023	0.0067	0.0003	−0.0010	0.0013	0.9
to S (0.5+x, 0.5−y, 0.5+z)							
O1···H21	1	0.0045	0.0168	0.0008	−0.0026	0.0034	−1.1
C2···H20	1	0.0045	0.0166	0.0011	−0.0020	0.0031	−1.1
C14···C21	1	0.0040	0.0115	0.0007	−0.0014	0.0021	−0.6
H3···C9	1	0.0000	0.0001	0.0000	0.0000	0.0000	3.1
C15···C22	1	0.0037	0.0117	0.0007	−0.0015	0.0022	−0.4
C16···Cl4	1	0.0044	0.0145	0.0008	−0.0019	0.0028	−1.0
Cl3···Cl4	1	0.0044	0.0141	0.0007	−0.0021	0.0028	−1.0
H2···H21	1	0.0025	0.0126	0.0011	−0.0010	0.0021	0.8
between 2D networks							
to T (0.5+x, 0.5−y, −0.5+z)							
H23···Cl1	1	0.0058	0.0194	0.0009	−0.0030	0.0039	−2.3
H17···Cl1	1	0.0047	0.0165	0.0009	−0.0024	0.0032	−1.2
N4···Cl1	1	0.0028	0.0085	0.0005	−0.0012	0.0017	0.5
to U (1+x, y, z)							
Cl3···H11	1	0.0074	0.0269	0.0013	−0.0041	0.0054	−3.8
to V (1−x, 1−y, 2−z)							
H11···H11	1	0.0016	0.0074	0.0006	−0.0006	0.0012	1.6
H5···Cl2	2	0.0078	0.0282	0.0012	−0.0046	0.0058	−4.1
H11···Cl2	2	0.0017	0.0060	0.0004	−0.0006	0.0011	1.6

[a] Number of equivalent bond paths (N_{equiv}). [b] Densities of all electrons ($\rho(r)$). [c] Laplacian of electron density ($\rho(r)$). [d] Energy density (H_b). [e] Potential energy density ($V(r)$). [f] Lagrangian kinetic energy ($G(r)$) (a.u.). [g] Interaction energies (E_{int}) (kJ/mol).

Table S7. *Cont.*

**1Br** at 90 K							
**Bond Path**	N_{equiv} [a]	$\rho(r)$ [b]	$\nabla^2\rho(r)$ [c]	H_b [d]	$V(r)$ [e]	$G(r)$ [f]	E_{int} [g]
within a 2D network							
to P (1−x, 1−y, 1−z)							
Br2···Br4	2	0.0101	0.0268	0.0004	−0.0058	0.0063	−6.3
C21···C21	1	0.0000	0.0002	0.0000	0.0000	0.0000	3.1
to Q (2−x, 1−y, 2−z)							
H15···C10,C11	2	0.0098	0.0342	0.0014	−0.0058	0.0072	−6.1
H14···O3	2	0.0024	0.0093	0.0006	−0.0010	0.0017	0.9
H14···C7	2	0.0034	0.0149	0.0010	−0.0016	0.0027	−0.0
Br2···Br3	2	0.0041	0.0113	0.0003	−0.0022	0.0025	−0.7
to R (1.5−x, −0.5+y, 1.5−z)							
N4···H9	1	0.0088	0.0320	0.0014	−0.0052	0.0066	−5.1
O1···H8	1	0.0106	0.0399	0.0015	−0.0069	0.0085	−6.8
H3···Br4	1	0.0080	0.0228	0.0005	−0.0048	0.0052	−4.3
H3···C22	1	0.0078	0.0261	0.0009	−0.0047	0.0056	−4.1
H17···Br2	1	0.0031	0.0107	0.0006	−0.0014	0.0021	0.2
Br1···Br4	1	0.0034	0.0096	0.0004	−0.0017	0.0020	−0.0
to S (0.5+x, 0.5−y, 0.5+z)							
O1···H21	1	0.0115	0.0389	0.0012	−0.0074	0.0086	−7.6
C2···H20	1	0.0034	0.0130	0.0009	−0.0014	0.0023	−0.1
C14···C21	1	0.0050	0.0150	0.0010	−0.0018	0.0028	−1.6
H3···C9	1	0.0001	0.0002	0.0000	0.0000	0.0000	3.0
C17···Br4	1	0.0065	0.0191	0.0008	−0.0032	0.0040	−2.9
Br3···Br4	1	0.0063	0.0163	0.0002	−0.0036	0.0038	−2.8
between 2D networks							
to T (0.5+x, 0.5−y, −0.5+z)							
H23···Br1	1	0.0078	0.0208	0.0004	−0.0044	0.0048	−4.2
H17···Br1	1	0.0046	0.0142	0.0006	−0.0024	0.0030	−1.2
N4···Br1	1	0.0031	0.0092	0.0004	−0.0014	0.0019	0.2
to U (1+x, y, z)							
Br3···H11	1	0.0102	0.0293	0.0006	−0.0060	0.0067	−6.4
Br3···H5	1	0.0026	0.0091	0.0005	−0.0012	0.0017	0.7
to V (1−x, 1−y, 2−z)							
H11···H11	1	0.0011	0.0049	0.0004	−0.0003	0.0008	2.1
H5···Br2	2	0.0099	0.0299	0.0008	−0.0059	0.0067	−6.1
Br1···Br2	2	0.0062	0.0156	0.0003	−0.0033	0.0036	−2.7

[a] Number of equivalent bond paths (N_{equiv}). [b] Densities of all electrons ($\rho(r)$). [c] Laplacian of electron density ($\rho(r)$). [d] Energy density (H_b). [e] Potential energy density ($V(r)$). [f] Lagrangian kinetic energy ($G(r)$) (a.u.). [g] Interaction energies (E_{int}) (kJ/mol).

Table S7. *Cont.*

			**1Br** at 373 K				
**Bond Path**	N_{equiv} [a]	$\rho(r)$ [b]	$\nabla^2\rho(r)$ [c]	H_b [d]	$V(r)$ [e]	$G(r)$ [f]	E_{int} [g]
within a 2D network							
to P (1−x, 1−y, 1−z)							
Br2···Br4	2	0.0097	0.0257	0.0004	−0.0056	0.0060	−6.0
C20···C20	1	0.0001	0.0003	0.0000	0.0000	0.0001	3.0
C9···H21	2	0.0018	0.0057	0.0004	−0.0006	0.0010	1.4
to Q (2−x, 1−y, 2−z)							
H15···C10	2	0.0113	0.0338	0.0009	−0.0066	0.0075	−7.4
H14···C8	2	0.0038	0.0145	0.0010	−0.0016	0.0026	−0.4
Br2···Br3	2	0.0030	0.0090	0.0004	−0.0015	0.0019	0.3
to R (1.5−x, −0.5+y, 1.5−z)							
N4···H9	1	0.0083	0.0291	0.0012	−0.0048	0.0061	−4.6
O1···H8	1	0.0095	0.0372	0.0016	−0.0061	0.0077	−5.8
H2···C20	1	0.0010	0.0035	0.0003	−0.0004	0.0006	2.1
H3···C22	1	0.0086	0.0278	0.0011	−0.0048	0.0059	−5.0
H17···Br2	1	0.0021	0.0082	0.0006	−0.0009	0.0015	1.1
Br1···Br4	1	0.0043	0.0116	0.0003	−0.0022	0.0026	−0.9
to S (0.5+x, 0.5−y, 0.5+z)							
O1···H21	1	0.0053	0.0187	0.0008	−0.0030	0.0038	−1.8
C2···H20	1	0.0026	0.0098	0.0007	−0.0010	0.0017	0.6
C14···C21	1	0.0030	0.0084	0.0005	−0.0010	0.0016	0.3
H3···C9	1	0.0000	0.0001	0.0000	0.0000	0.0000	3.1
C16···Br4	1	0.0047	0.0138	0.0006	−0.0023	0.0029	−1.3
Br3···Br4	1	0.0043	0.0122	0.0003	−0.0024	0.0027	−0.9
Br1···C11	1	0.0000	0.0001	0.0000	0.0000	0.0000	3.1
between 2D networks							
to T (0.5+x, 0.5−y, −0.5+z)							
H23···Br1	1	0.0042	0.0124	0.0005	−0.0020	0.0026	−0.8
H17···Br1	1	0.0066	0.0190	0.0005	−0.0037	0.0042	−3.1
Br3···Br1	1	0.0027	0.0083	0.0004	−0.0014	0.0017	0.6
to U (1+x, y, z)							
Br3···H11	1	0.0084	0.0266	0.0009	−0.0048	0.0057	−4.7
to V (1−x, 1−y, 2−z)							
H11···H11	1	0.0015	0.0070	0.0006	−0.0005	0.0012	1.7
H5···Br2	2	0.0065	0.0197	0.0007	−0.0036	0.0043	−2.9
Br1···Br2	2	0.0033	0.0096	0.0003	−0.0017	0.0020	−0.0
H11···Br2	2	0.0021	0.0078	0.0005	−0.0009	0.0014	1.2

[a] Number of equivalent bond paths (N_{equiv}). [b] Densities of all electrons ($\rho(r)$). [c] Laplacian of electron density ($\rho(r)$). [d] Energy density (H_b). [e] Potential energy density ($V(r)$). [f] Lagrangian kinetic energy ($G(r)$) (a.u.). [g] Interaction energies (E_{int}) (kJ/mol).

Table S7. *Cont.*

Bond Path	N_{equiv} [a]	$\rho(r)$ [b]	$\nabla^2\rho(r)$ [c]	H_b [d]	$V(r)$ [e]	$G(r)$ [f]	E_{int} [g]
**1I** at 90 K							
within a 2D network							
to P (2−x, 1−y, 1−z)							
N2···H21	2	0.0112	0.0388	0.0013	−0.0072	0.0084	−7.3
O4···H20	2	0.0069	0.0261	0.0011	−0.0042	0.0054	−3.4
H20···H20	1	0.0043	0.0211	0.0016	−0.0022	0.0037	−0.9
H11···I4	2	0.0045	0.0172	0.0011	−0.0021	0.0032	−1.1
to Q (x, 1−y, 0.5+z)							
H3···C24	1	0.0101	0.0314	0.0010	−0.0059	0.0069	−6.4
C17···H9	1	0.0074	0.0277	0.0014	−0.0041	0.0055	−3.8
N4···H9	1	0.0076	0.0264	0.0011	−0.0045	0.0056	−4.0
O1···H8	1	0.0046	0.0189	0.0012	−0.0024	0.0036	−1.2
H23···I2	1	0.0067	0.0253	0.0014	−0.0035	0.0049	−3.1
I1···C22	1	0.0060	0.0201	0.0012	−0.0027	0.0039	−2.5
H2···O2	1	0.0031	0.0118	0.0006	−0.0017	0.0023	0.2
H2···O4	1	0.0021	0.0086	0.0006	−0.0010	0.0016	1.1
H2···C8	1	0.0027	0.0115	0.0009	−0.0011	0.0020	0.6
I4···I2	1	0.0026	0.0109	0.0009	−0.0010	0.0018	0.7
to R (1−x, y, 0.5−z)							
H8···H14	2	0.0050	0.0236	0.0017	−0.0026	0.0042	−1.6
H8···H15	2	0.0069	0.0279	0.0013	−0.0044	0.0057	−3.3
H15···O2	2	0.0050	0.0211	0.0013	−0.0027	0.0040	−1.6
C13···C14	2	0.0029	0.0087	0.0006	−0.0010	0.0016	0.4
C15···C18	2	0.0028	0.0083	0.0005	−0.0010	0.0016	0.5
C16···C17	2	0.0028	0.0080	0.0005	−0.0010	0.0015	0.5
N4···I3	2	0.0020	0.0078	0.0006	−0.0008	0.0014	1.2
to S (1−x, 1−y, 1−z)							
C3···I3	2	0.0041	0.0146	0.0010	−0.0016	0.0026	−0.7
C2···H15	2	0.0022	0.0071	0.0005	−0.0008	0.0013	1.0
C14···C14	1	0.0019	0.0051	0.0003	−0.0006	0.0009	1.3
between 2D networks							
to T (1.5−x, 1.5−y, 1−z)							
I3···I4	2	0.0080	0.0297	0.0013	−0.0048	0.0061	−4.3
H23···H23	1	0.0000	0.0001	0.0000	0.0000	0.0000	3.1
to U (1.5−x, 0.5−y, −z)							
I2···I2	1	0.0094	0.0334	0.0012	−0.0059	0.0071	−5.6
to V (−0.5+x, 0.5+y, z)							
I3···I2	1	0.0010	0.0044	0.0004	−0.0002	0.0007	2.2

[a] Number of equivalent bond paths (N_{equiv}). [b] Densities of all electrons ($\rho(r)$). [c] Laplacian of electron density ($\rho(r)$). [d] Energy density (H_b). [e] Potential energy density ($V(r)$). [f] Lagrangian kinetic energy ($G(r)$) (a.u.). [g] Interaction energies (E_{int}) (kJ/mol).

Table S7. *Cont.*

**1I** at 373 K							
**Bond Path**	N_{equiv} [a]	$\rho(r)$ [b]	$\nabla^2\rho(r)$ [c]	H_b [d]	$V(r)$ [e]	$G(r)$ [f]	E_{int} [g]
within a 2D network							
to P (2−x, 1−y, 1−z)							
N2···H21	2	0.0113	0.0372	0.0011	−0.0071	0.0082	−7.4
O4···H20	2	0.0033	0.0126	0.0007	−0.0018	0.0025	0.0
H20···H20	1	0.0038	0.0182	0.0013	−0.0019	0.0032	−0.4
H11···I4	2	0.0019	0.0077	0.0007	−0.0006	0.0012	1.4
to Q (x, 1−y, 0.5+z)							
H3···C24	1	0.0084	0.0273	0.0012	−0.0045	0.0056	−4.7
C17···H9	1	0.0049	0.0208	0.0013	−0.0025	0.0039	−1.5
N4···H9	1	0.0123	0.0382	0.0009	−0.0077	0.0086	−8.4
O1···H8	1	0.0058	0.0234	0.0013	−0.0033	0.0046	−2.3
H23···I2	1	0.0044	0.0182	0.0013	−0.0020	0.0033	−1.0
I1···C22	1	0.0043	0.0155	0.0010	−0.0018	0.0028	−0.9
H2···O2	1	0.0013	0.0053	0.0004	−0.0006	0.0010	1.9
H2···O4	1	0.0027	0.0102	0.0006	−0.0014	0.0020	0.6
I1···I4	1	0.0064	0.0248	0.0013	−0.0035	0.0049	−2.8
to R (1−x, y, 0.5−z)							
H8···H15	2	0.0024	0.0113	0.0009	−0.0011	0.0020	0.9
H15···O2	2	0.0028	0.0119	0.0008	−0.0013	0.0022	0.5
H14···O2	2	0.0028	0.0115	0.0008	−0.0013	0.0021	0.5
C13···C14	2	0.0024	0.0074	0.0005	−0.0009	0.0014	0.9
C16···C17	2	0.0029	0.0082	0.0005	−0.0010	0.0015	0.4
to S (1−x, 1−y, 1−z)							
C4···I3	2	0.0043	0.0166	0.0011	−0.0019	0.0030	−0.9
C2···H15	2	0.0012	0.0036	0.0003	−0.0004	0.0006	2.0
C14···C14	2	0.0018	0.0049	0.0003	−0.0006	0.0009	1.5
I1···I3	2	0.0039	0.0168	0.0011	−0.0019	0.0031	−0.5
between 2D networks							
to T (1.5−x, 1.5−y, 1−z)							
I3···I4	2	0.0063	0.0252	0.0013	−0.0037	0.0050	−2.8
H23···H23	1	0.0000	0.0001	0.0000	0.0000	0.0000	3.1
to U (1.5−x, 0.5−y, −z)							
I2···I2	1	0.0066	0.0261	0.0013	−0.0038	0.0052	−3.0
to V (−0.5+x, 0.5+y, z)							
I3···I2	1	0.0007	0.0029	0.0003	−0.0001	0.0004	2.5

[a] Number of equivalent bond paths (N_{equiv}). [b] Densities of all electrons ($\rho(r)$). [c] Laplacian of electron density ($\rho(r)$). [d] Energy density (H_b). [e] Potential energy density ($V(r)$). [f] Lagrangian kinetic energy ($G(r)$) (a.u.). [g] Interaction energies (E_{int}) (kJ/mol).

Table S8. Properties (a.u.) of the bond critical points (BCPs) along the bond paths for electron density distribution of molecular pairs of the $[Fe(\mathbf{L}^{Br})_2]^-$ anion with L1' and L2 and interaction energies (kJ/mol) calculated from the density of all electrons at the BCPs in **1Br** at 90 K.

**1Br** at 90 K							
**Bond Path**	N_{equiv} [a]	$\rho(r)$ [b]	$\nabla^2\rho(r)$ [c]	H_b [d]	$V(r)$ [e]	$G(r)$ [f]	E_{int} [g]
within a 2D network							
to P (1−x, 1−y, 1−z)							
Br1′···Br4	2	0.0077	0.0204	0.0004	−0.0043	0.0047	−4.0
C21···C21	1	0.0000	0.0002	0.0000	0.0000	0.0000	3.1
C3'···Br4	2	0.0067	0.0209	0.0009	−0.0034	0.0043	−3.1
to Q (2−x, 1−y, 2−z)							
H15···C4'	2	0.0101	0.0341	0.0012	−0.0061	0.0073	−6.4
H14···C1'	2	0.0039	0.0171	0.0012	−0.0019	0.0031	−0.6
O3···H14	2	0.0023	0.0093	0.0006	−0.0010	0.0017	0.9
Br3···Br1'	2	0.0047	0.0125	0.0003	−0.0025	0.0028	−1.3
to R (1.5−x, −0.5+y, 1.5−z)							
N4···H3'	1	0.0070	0.0252	0.0012	−0.0039	0.0051	−3.4
O2'···H2'	1	0.0117	0.0423	0.0014	−0.0078	0.0092	−7.8
H9'···C22	1	0.0109	0.0342	0.0010	−0.0064	0.0075	−7.0
H17···Br1'	1	0.0010	0.0036	0.0003	−0.0003	0.0006	2.2
Br2'···Br4	1	0.0069	0.0167	0.0002	−0.0037	0.0039	−3.4
to S (0.5+x, 0.5−y, 0.5+z)							
O2'···H21	1	0.0065	0.0222	0.0009	−0.0038	0.0047	−2.9
C8'···H20	1	0.0041	0.0171	0.0012	−0.0018	0.0031	−0.8
C14···C21	1	0.0051	0.0151	0.0010	−0.0018	0.0028	−1.6
H8′···H21	1	0.0056	0.0258	0.0016	−0.0032	0.0048	−2.2
C17···Br4	1	0.0065	0.0191	0.0008	−0.0032	0.0040	−3.0
Br3···Br4	1	0.0063	0.0163	0.0002	−0.0036	0.0038	−2.8
Br2'···Br1'	1	0.0001	0.0005	0.0000	0.0000	0.0001	3.1
H9'···C3'	1	0.0000	0.0002	0.0000	0.0000	0.0000	3.1
between 2D networks							
to T (0.5+x, 0.5−y, −0.5+z)							
H23···Br2'	1	0.0060	0.0166	0.0005	−0.0032	0.0037	−2.5
H17···Br2'	1	0.0028	0.0099	0.0006	−0.0013	0.0019	0.5
to U (1+x, y, z)							
Br3···H11	1	0.0028	0.0098	0.0006	−0.0013	0.0019	0.5
Br3···H5'	1	0.0115	0.0332	0.0006	−0.0070	0.0077	−7.6
Br3···Br1'	1	0.0030	0.0092	0.0004	−0.0015	0.0019	0.3
to V (1−x, 1−y, 2−z)							
Br1'···H11'	2	0.0076	0.0234	0.0008	−0.0043	0.0051	−6.1
H5'···Br1'	2	0.0022	0.0083	0.0006	−0.0010	0.0015	1.1
Br1'···N2'	2	0.0031	0.0097	0.0005	−0.0015	0.0020	0.2

[a] Number of equivalent bond paths (N_{equiv}). [b] Densities of all electrons ($\rho(r)$). [c] Laplacian of electron density ($\rho(r)$). [d] Energy density (H_b). [e] Potential energy density ($V(r)$). [f] Lagrangian kinetic energy ($G(r)$) (a.u.). [g] Interaction energies (E_{int}) (kJ/mol).
